# Impact of polygeneration topology on the technoeconomic performance of green hydrogen production utilizing integrated CPVT systems

**DOI:** 10.1038/s41598-026-63619-4

**Published:** 2026-08-06

**Authors:** Khaled Darwish, Adel Khalil, Ahmed Aboulmagd

**Affiliations:** 1 Mechanical Power Engineering Department, Faculty of Engineering, Cairo University, Giza, 12613 Egypt; 2 Mechanical Engineering Program, School of Engineering and Applied Sciences, Nile University, Giza, 12677 Egypt

**Keywords:** Polygeneration, CPVT, Triple-junction, PEM electrolysis, MEE desalination, Green hydrogen, LCOH, Energy science and technology, Engineering

## Abstract

This paper presents a year-long hour-by-hour dynamic comparative analysis of solar-driven polygeneration configurations at three Egyptian Sea coast locations — Hurghada, Suez, and Port Said. A MATLAB/SIMSCAPE computational framework is developed and validated, coupling a concentrating parabolic trough collector (PTC) to two receiver technologies: a conventional concentrating photovoltaic-thermal (CPVT), receiver-A, and a high-efficiency GaInP/GaInAs/Ge triple-junction photovoltaic-thermal (C3JT), receiver-B. Both are integrated with proton exchange membrane (PEM) electrolyzer and multi-effect evaporation (MEE) desalination units. Four configurations are evaluated: Polygeneration-I (Cases I-A and I-B) producing hydrogen and freshwater, and Polygeneration-II (Cases II-A and II-B) producing hydrogen and exporting electricity. The results show that the C3JT architecture consistently outperforms CPVT by 57.1–57.7% in annual hydrogen production across all configurations and locations. A global minimum levelized cost of hydrogen (LCOH) of 2.909 USD/kg is achieved by Case II-B at Hurghada, representing a 32.6% reduction relative to the Polygeneration-I CPVT baseline (Case I-A) at the same location. MEE thermal integration introduces an energy partitioning trade-off that reduces system-to-hydrogen HHV efficiency to 4.53–7.43% across Polygeneration-I configurations, yet this is more than offset by freshwater revenue credits in the economic framework. The results also demonstrate the economic viability of concentrating solar polygeneration in Egypt without requiring gigawatt-scale deployment.

## Introduction

The global energy sector remains the dominant source of anthropogenic greenhouse gas emissions, accounting for over three-quarters of total annual CO_2_ output^1^. Decarbonizing this sector demands a fundamental shift toward clean, renewable energy carriers, among which green hydrogen has emerged as a solution due to its high energy density, near zero-emission, and compatibility with existing industrial infrastructure. In sunbelt regions, which are broadly defined as the latitudinal band between 15° N and 40° N where annual direct normal irradiance (DNI) typically exceeds 1700 kWh m^− 2^ yr^− 1^, where solar irradiance is abundant and fossil fuel dependence remains high, solar-driven hydrogen production presents a critical pathway. Egypt occupies a favorable position in this regard: its Red Sea coastal cities benefit from among the highest direct normal irradiance (DNI) resources in the world, as demonstrated by Ziyaei et al.^2^, who identified Hurghada as achieving the highest solar energy share among all cities in their global comparative desalination study, confirming the exceptional solar resource available along Egypt’s Red Sea coast. Egypt simultaneously confronts a deepening water crisis. Freshwater availability across the Middle East and North Africa (MENA) region is projected to decline by 2050 under current scenarios, with non-renewable groundwater reserves at risk of depletion within the same timeframe^3^. Seawater desalination is an energy intensive process, such as in multi-effect evaporation (MEE) which consumes significant energy per kilogram of produced water^4^. An average value of the specific energy consumption is calculated as 230 kJ/kg per stage of a typical MEE system. But when the driving energy is solar-derived and desalination is integrated as a co-production output alongside hydrogen, net economics can be strongly favorable.

Concentrating photovoltaic-thermal (CPVT) technology is uniquely positioned to serve such integrated functions. By focusing solar irradiance onto high-efficiency photovoltaic flat receivers, CPVT systems simultaneously produce electrical and thermal energy from a single solar collection aperture. Triple-junction (C3JT) solar cells have demonstrated exceptional conversion efficiencies under concentrated illumination: lattice-matched GaInP/GaInAs/Ge concentrator cells were the first photovoltaic devices of any type to surpass the 40% efficiency milestone, reaching 40.7%^5^. This spectral efficiency advantage arises from current matching across three series-connected sub-cells that collectively harvest a broader portion of the solar spectrum than any single-junction device^6,7^, making C3JT cells the technology of choice for high-concentration solar hydrogen systems. When coupled to a proton exchange membrane (PEM) electrolyzer, which offers high current density, rapid dynamic response, and compatibility with intermittent solar power^8^, the resulting integrated system can produce hydrogen at competitive levelized costs.

The case for solar hydrogen production in the MENA region has received growing research attention. Al-Sharafi et al.^9^ reported hydrogen costs of 39.5–43.1 USD/kg for off-grid solar-wind hybrid systems at five Saudi Arabian locations using HOMER optimization, reflecting the early economics of small isolated systems without co-product integration. More recent large-scale analyses have shown dramatically improved economics: Muhieitheen et al.^10^ assessed a hybrid PV-wind polygeneration system in Saudi Arabia, reporting an LCOH below 1.059 USD/kg when excess renewable electricity drove electrolysis alongside a levelized water cost of 2.16–2.28 USD/m^3^. Al-Ghussain et al.^11^ demonstrated an LCOH of 1.61 USD/kg for a country-scale 100% renewable energy system in Jordan, combining excess generation for hydrogen production with reverse osmosis desalination at 0.36 USD/m^3^. These studies establish that multi-output co-production is not only technically feasible but economically advantageous when all revenue streams are credited.

Beyond the MENA region, recent studies have further advanced the techno-economic and thermodynamic assessment of renewable-driven polygeneration systems. Shokri Kalan et al.^12^ proposed an integrated solar–geothermal polygeneration system on Tenerife Island combining a supercritical CO₂ cycle, three-stage ORC, LiBr absorption cooling, MEE desalination, and PEM electrolysis for simultaneous production of power, heating, cooling, freshwater, and hydrogen, achieving baseline energy and exergy efficiencies of 62% and 17%, respectively, with grey wolf optimization improving hydrogen output by 18% and Monte Carlo simulations quantifying the sensitivity of system outputs to DNI variability. In a complementary methodological direction, Babaei Khuyinrud et al.^13^ applied advanced exergy and exergoeconomic analyses to a solar-driven Kalina cycle with molten-salt storage under variable load conditions, demonstrating that component-level decomposition of avoidable and unavoidable exergy destruction can identify targeted cost-reduction pathways — reducing electricity production cost by 8.45% through multi-objective optimization. While these studies demonstrate the breadth of analytical frameworks now available for solar polygeneration assessment, none has performed a year-long hourly dynamic comparison of concentrating PV receiver technologies across multiple polygeneration topologies under real site-specific meteorological conditions, which is the contribution of the present work.

Solar thermal desalination integration has been explored across multiple technology pathways. Ziyaei et al.^2^ performed a dynamic life cycle cost analysis of solar parabolic trough-driven multi-stage flash desalination across seven global cities, finding that Hurghada, Egypt achieved the highest solar energy share and the lowest levelized cost of desalinated water (2.21 USD/m^3^) among all locations studied, these results directly motivate the selection of Egyptian Red Sea coast cities in the present analysis. Pouyfaucon and García-Rodríguez^4^ assessed the market positioning of solar thermal desalination technologies, noting that multi-effect evaporation distillation combined with a double-effect absorption heat pump achieves a performance ratio of up to 20 but requires careful thermal integration with the driving solar system. Anand and Murugavelh^14^ demonstrated a CPVT trigeneration system coupling a triple-junction receiver to humidification-dehumidification desalination and vapor compression cooling, achieving a payback period of 6.5 years and confirming that multi-output CPVT architectures are economically viable when all co-product revenues are accounted for.

The electrical and thermal behavior of triple-junction cells under concentrated illumination has been characterized in the literature. Theristis and O’Donovan^15^ developed an integrated electrical-thermal model for GaInP/GaInAs/Ge cells at a concentration ratio of 500x, demonstrating that without active cooling the cell temperature can exceed 1,000 °C and that a thermal resistance below 1.63 K/W is required for safe passive operation. *Helmers et al.*^16^ measured open-circuit voltage temperature coefficients for individual sub-cells over different concentration range and temperatures, providing the analytical open voltage and temperature relationship essential for coupled electrical-thermal simulation. Nishioka et al.^17^ showed experimentally that the normalized temperature coefficient of efficiency improves from − 0.248%/°C at 1 sun to − 0.098%/°C at 200 suns, supporting the use of high-concentration architectures in hot climates. Roensch et al.^18^ extracted individual sub-cell I-V characteristics by electroluminescence measurements, confirming that the Ge bottom sub-cell exhibits near-ideal diode behavior while the GaInP and GaInAs sub-cells show Shockley-Read-Hall recombination informing the double-diode parameterization adopted in this work.

Several researchers have explored CPVT system configurations for multi-output applications. Riahi et al.^19^ integrated thermoelectric generators with a CPVT prototype in Tunisia, achieving a 7.46% improvement in daily electrical efficiency through thermal recovery augmentation and validating a coupled electrical-thermal model against experimental prototype data. Alayi et al.^20^ performed energy, economic, and environmental analyses of parabolic trough CPVT systems across four Iranian cities, demonstrating that high-solar-resource locations achieve substantially greater combined thermal and electrical outputs. Acosta-Pazmiño et al.^21^ evaluated an LCPV/T plant in Mexico using TRNSYS dynamic simulation, proposing a levelized cost of heat framework that demonstrated near parity with natural gas boiler economics under local pricing conditions. On the modelling side, Da Silva and Fernandes^22^ developed a modular SIMULINK strategy for PV/T systems, the Suskis and Galkin^23^ enhanced SIMSCAPE photovoltaic block incorporating junction capacitance effects for improved dynamic accuracy, and the cell-to-array scaling framework of Tian et al.^24^ together with the meteorological parameter integration methodology of Nfaoui et al.^25^, form the computational foundations of the simulation platform employed in the present study.

Despite this previous work, a critical gap remains. To the authors’ knowledge, the polygeneration system proposed in the present study — integrating a CPVT/C3JT concentrating receiver with PEM electrolysis and MEE thermal desalination — has not been reported in the existing literature. The comparative impact of progressively expanding from a hydrogen-only architecture to full polygeneration that integrates MEE thermal desalination and electricity export as successive outputs, has not been identified. Furthermore, no study, to the authors’ knowledge, has performed a year-long dynamic comparison of CPVT and C3JT concentrating architectures across this full spectrum at multiple Egyptian sites characterized by real DNI, ambient temperature, wind speed and seawater salinity data. The present study addresses this gap by developing a MATLAB /SIMSCAPE dynamic simulation model that integrates all subsystems such as concentrating solar collector (parabolic trough), CPVT or C3JT flat receiver, PEM electrolyzer and MEE desalination, and applies it to different polygeneration configurations at Hurghada, Suez, and Port Said. The central research questions are: (i) Which receiver technology delivers superior annual hydrogen production and system efficiency across all polygeneration configurations? (ii) Which polygeneration architecture achieves lower LCOH? and (iii) Which combination of receiver technology, configuration, and Egyptian location achieves the global minimum LCOH?

The remainder of this paper is organized as follows. “System description and mathematical modelling” presents the system architecture, geometric parameters, and complete mathematical models for all subsystems.   “Simulation methodology and environment” describes the simulation methodology and meteorological input data. “Results and discussions” presents and discusses results across all four configurations at three locations.   “Conclusions” draws conclusions.

## System description and mathematical modelling

### System topology descriptions

**Fig. 1:**
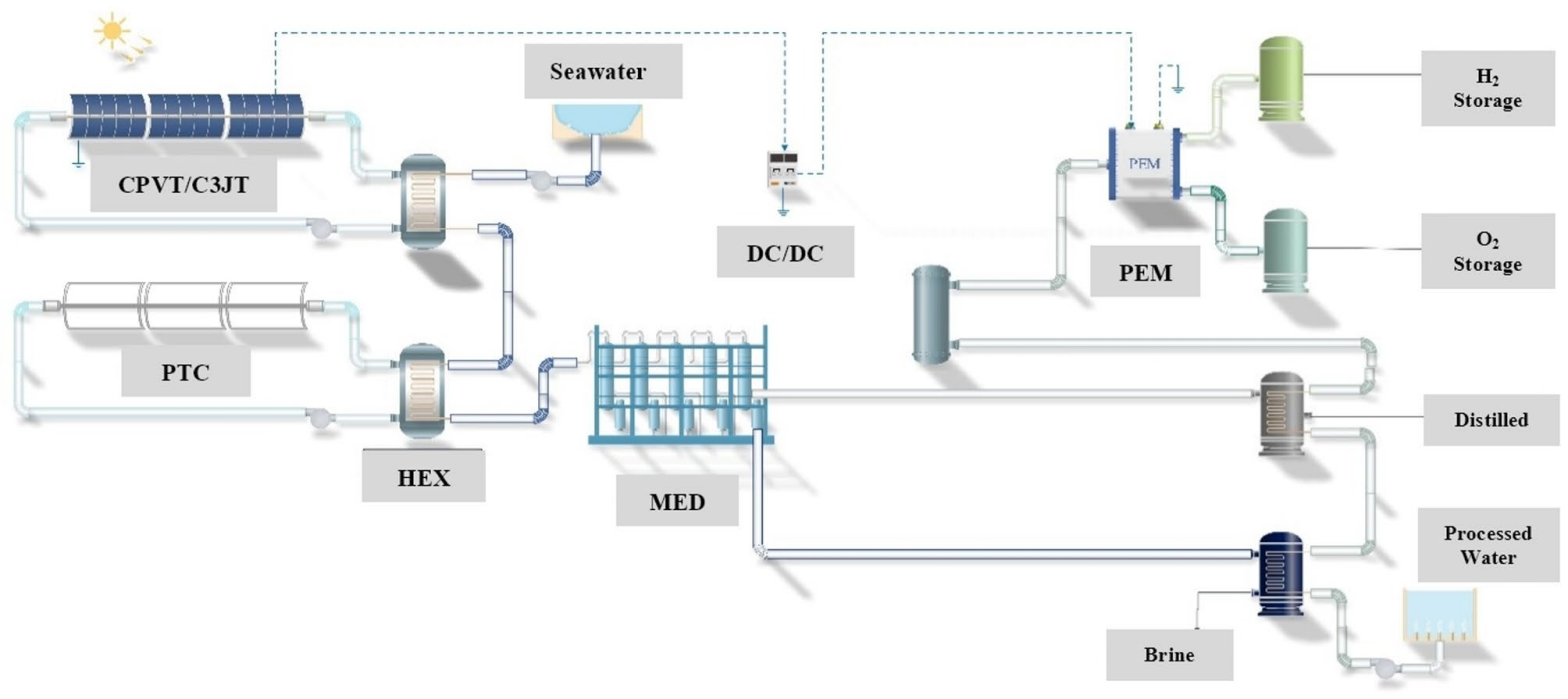
Schematic of the integrated concentrating solar polygeneration-I system.

**Fig. 2:**
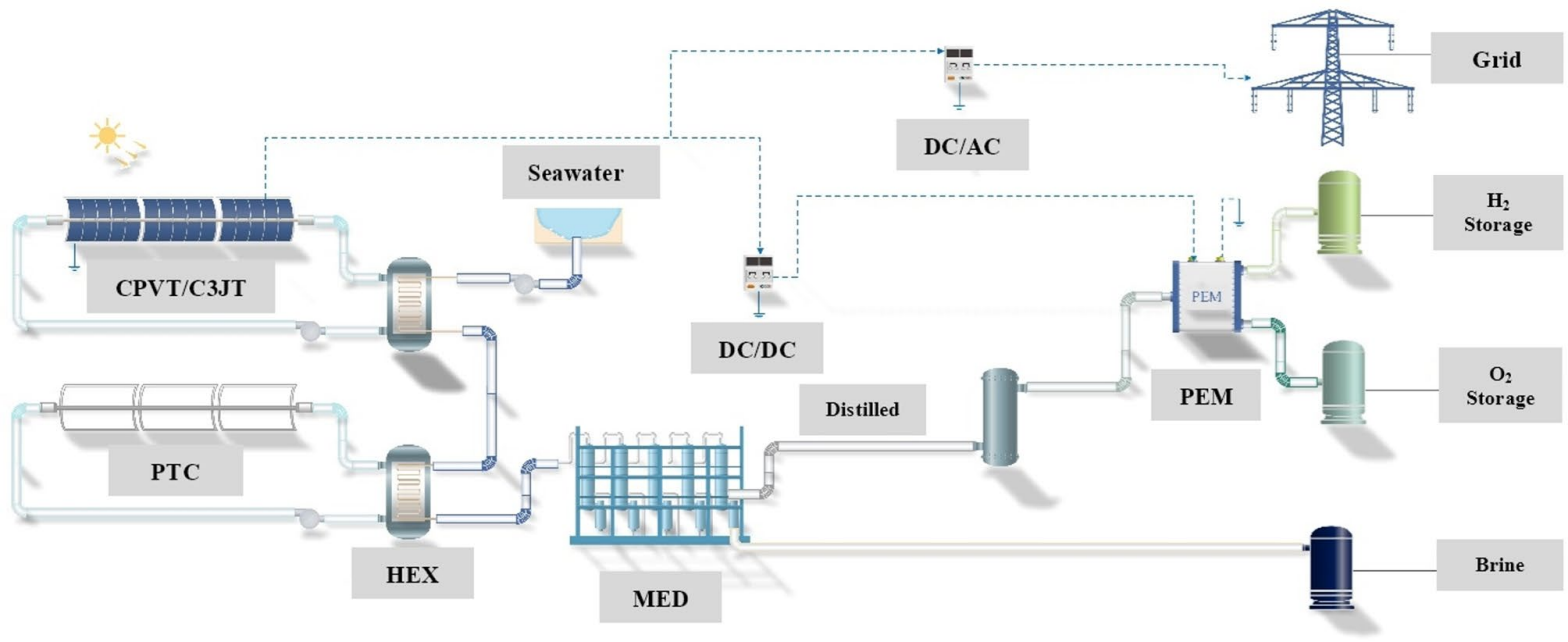
Schematic of the integrated concentrating solar polygeneration-II system.

The integrated concentrating solar polygeneration system consists of four interconnected subsystems: (1) a parabolic trough solar concentrating collector with a flat thermal receiver available in two technology variants (CPVT / C3JT); (2) a parabolic trough solar concentrating collector with a flat thermal receiver for thermal heating (PTC); (3) a proton exchange membrane electrolyzer for green hydrogen production; and (4) a multi-effect evaporation desalination unit (MEE). Figures 1 and 2 show the system schematic for polygeneration-I and polygeneration-II. The system is designed for distributed deployment at Egyptian Sea coast locations with all thermal energy co-products directed to the desalination unit.

For Polygeneration-I, the system operates as a dual output polygeneration plant. The parabolic trough collector delivers concentrated solar flux to the receiver, which simultaneously generates electrical power and extracts useful heat through the coolant loop. The full electrical output is routed to the PEM electrolyzer that receives external deionized water as feedstock and consumes the full available electrical output of the receiver to produce hydrogen. The MEE unit operates in parallel, independently consuming the receiver thermal output to produce freshwater. The thermal output is directed to the MEE desalination unit, where it serves as the motive for heat source driving successive evaporation effects. Both hydrogen and freshwater are produced continuously during daylight hours, with production rates governed by the instantaneous solar resource at each location.

For Polygeneration-II, the MEE subsystem operates identically to polygeneration-I except that the distillated produced by the MEE unit is fed directly into the PEM electrolyzer as the hydrogen production feedstock. At each hourly timestep, the electrical power required to electrolyze the MEE distillate flow rate is computed and the remaining electrical power is exported. The hydrogen production rate is therefore controlled by the MEE distillate output.

Two receiver technology variants are evaluated in parallel, defining the A/B subscript throughout the case naming:

CPVT receiver (Cases A): a linear parabolic trough concentrating collector focuses direct normal irradiance onto a flat receiver incorporating silicon-based multi-crystalline photovoltaic cells laminated to the absorber surface. A single-pass water coolant loop extracts waste heat from the receiver, maintaining cell temperatures at acceptable operating levels while simultaneously generating usable thermal output. The geometrical concentration ratio of 32x is moderate, consistent with established parabolic trough CPVT designs. Multi-crystalline silicon cells were selected instead of mono-crystalline cells for cost reasons. At a low concentration ratio of 32x, the system performance is mainly limited by optical and thermal losses rather than by cell efficiency, so the lower cost of multi-crystalline silicon makes it the more practical choice for a distributed-scale system.

C3JT receiver (Cases B): the same parabolic trough geometry is equipped with a high-efficiency GaInP/GaInAs/Ge triple-junction receiver operating at the same geometric concentration ratio. The triple-junction cell exploits current matching across three series-connected sub-cells, capturing a substantially broader portion of the solar spectrum than any single-junction device.

### Parabolic trough collector optical model

The solar collection subsystem is a single-axis north-south tracking parabolic trough. The aperture area and incident solar power are determined by the collector geometry. The key geometric and optical parameters are given in Table 1 for both receiver variants. The instantaneous optical power delivered to the receiver is:

1 $$\:{Q}_{\mathrm{o}\mathrm{p}\mathrm{t}}\left(t\right)={\eta\:}_{\mathrm{o}\mathrm{p}\mathrm{t}}\left(\theta\:\right)\cdot \:DNI\left(t\right)\cdot \:{A}_{\mathrm{a}\mathrm{p}}$$

where DNI(t) is the direct normal irradiance (W m^− 2^) and A_ap_ is the aperture area of the PTC.

The overall optical efficiency of the concentrating system, $\:{\eta\:}_{\mathrm{o}\mathrm{p}\mathrm{t}}\left(\theta\:\right)$, accounts for mirror reflectivity ρ, cover transmissivity τ, receiver absorptivity α, incidence angle modifier IAM(θ), and intercept factor γ and is given by:

2 $$\:{\eta\:}_{opt}=\rho\:\cdot\:\tau\:\cdot\:\alpha\:\cdot\:\gamma\:\cdot\:\mathrm{I}\mathrm{A}\mathrm{M}\left({\uptheta\:}\right)$$

The geometric concentration ratio $\:{CR}_{g}$ relates the aperture width (W) to the receiver width (w) and is given by:

3 $$\:{CR}_{g}=\frac{{W}_{aperature}}{{w}_{receiver}}$$

**Table 1:** Parabolic trough collector and receiver parameters for both receiver technologies.

Parameter	Value
Aperture width (m)	5
Collector length (m)	8
Number of collectors	6
Focal length (m)	1.489
Rim angle (deg)	80
Geometric concentration ratio	32
Overall optical efficiency	0.92
Rectangular channel height (m)	0.05
Rectangular channel width (m)	0.156
Rectangular channel thickness (mm)	3

### Concentrated photovoltaic model

The CPVT receiver photovoltaic output is computed using a double-diode equivalent circuit, which is the standard framework for silicon-based concentrating PV cells and has been validated in CPVT system dynamic simulations^19^. The current-voltage equation at cell temperature T_c_ and irradiance G is^7,23,24^:

4 $$\:I={I}_{ph}-{I}_{01}\left[\mathrm{e}\mathrm{x}\mathrm{p}\left(\frac{V+I{R}_{s}}{{n}_{1}{V}_{t}}\right)-1\right]-{I}_{02}\left[\mathrm{e}\mathrm{x}\mathrm{p}\left(\frac{V+I{R}_{s}}{{n}_{2}{V}_{t}}\right)-1\right]-\frac{V+I{R}_{s}}{{R}_{sh}}$$

where, *\:I* is the cell current, $\:{I}_{ph}$ is the photocurrent, $\:{I}_{0,i}$ is the diode saturation current,, *\:V* is the cell voltage, $\:{R}_{s}$ and $\:{R}_{sh}$ are the series and shunt resistances, $\:{n}_{i}$ is the diode ideality factor ($\:{n}_{1}=1.5$, $\:{n}_{2}=2)$, $\:{V}_{t}$ is thermal voltage at cell temperature and equals to $\:\frac{k\:{T}_{c}}{q}$, *\:k* is Boltzmann’s constant, $\:{T}_{c}$ is the cell temperature, *\:q* is the electron charge, $\:{I}_{sc,ref}$ is the reference short-circuit current at standard test conditions and $\:{\alpha\:}_{Isc}$ is the temperature coefficient of short-circuit current.

The photogenerated current is corrected for irradiance deviation from standard test conditions ($\:{DNI}_{ref}\:$= 1,000 W m^− 2^, $\:{T}_{ref}$ = 25 °C) and for temperature^24^:

5 $$\:{I}_{ph}=\left[{I}_{sc,ref}+{\alpha\:}_{Isc}\left({T}_{c}-{T}_{ref}\right)\right]\cdot\:{CR}_{g}\cdot\:\frac{\mathrm{D}\mathrm{N}\mathrm{I}}{{DNI}_{ref}}$$

where $\:{I}_{sc,ref}$ is the reference short-circuit current at standard test conditions and $\:{\alpha\:}_{Isc}$ is the temperature coefficient of short-circuit current.

The diode reverse saturation current is the most temperature-sensitive parameter in the model and is described as follows^24^:

6 $$\:{I}_{0,i}\left({T}_{c}\right)={I}_{0,\mathrm{\:ref,}i}{\left(\frac{{T}_{c}}{{T}_{\mathrm{ref\:}}}\right)}^{3}\mathrm{e}\mathrm{x}\mathrm{p}\left[\frac{{E}_{g}\left({T}_{\mathrm{ref\:}}\right)-{E}_{g}\left({T}_{c}\right)}{{n}_{i}\cdot\:k}\left(\frac{1}{{T}_{\mathrm{ref\:}}}-\frac{1}{{T}_{c}}\right)\right]$$

where, $\:{I}_{0,\mathrm{\:ref,}i}\:$is the saturation current at reference temperature, $\:{E}_{g}\left({T}_{c}\right)$ is the semiconductor bandgap energy at cell temperature (E_g,0 K_ = 1.12 eV) and $\:{T}_{ref}$ is the reference temperature in kelvin.

### Triple-junction photovoltaic model

The C3JT receiver employs a double-diode model for each of three series-connected sub-cells (j = 1,2 and 3, where top is 1; GaInP, middle is 2; GaInAs and bottom is 3; Ge), following the approach and validated against the experimental datasets of Helmers et al.^16^ and under concentration validation against Catelani et al.^7^. The double-diode formulation for sub-cell (j) is:

7 $$\:{I}_{\boldsymbol{j}}={I}_{ph,\boldsymbol{j}}-{I}_{01,\boldsymbol{j}}\left[\mathrm{e}\mathrm{x}\mathrm{p}\left(\frac{{V}_{\boldsymbol{j}}+{I}_{\boldsymbol{j}}{R}_{s,\boldsymbol{j}}}{{n}_{1,\boldsymbol{j}}{V}_{t,\boldsymbol{j}}}\right)-1\right]-{I}_{02,\boldsymbol{j}}\left[\mathrm{e}\mathrm{x}\mathrm{p}\left(\frac{{V}_{\boldsymbol{j}}+{I}_{\boldsymbol{j}}{R}_{s,\boldsymbol{j}}}{{n}_{2,\boldsymbol{j}}{V}_{t,\boldsymbol{j}}}\right)-1\right]-\frac{{V}_{\boldsymbol{j}}+{I}_{\boldsymbol{j}}{R}_{s,\boldsymbol{j}}}{{R}_{sh,\boldsymbol{j}}}$$

with,

8 $$\:{I}_{ph,\boldsymbol{j}}=\left[{I}_{sc,\boldsymbol{j},ref}+{\alpha\:}_{Isc,\boldsymbol{j}}\left({T}_{c}-{T}_{ref}\right)\right]\cdot\:{CR}_{g}\cdot\:{f}_{\boldsymbol{j}}\cdot\:\frac{\mathrm{D}\mathrm{N}\mathrm{I}}{1000}$$

where $\:{n}_{1,\boldsymbol{j}}$ and $\:{n}_{2,\boldsymbol{j}}$ is the diode ideality factor at each sub-cell. For top cell $\:{n}_{\mathrm{1,1}}=1.5$ and $\:{n}_{\mathrm{2,1}}=2$, mid cell $\:{n}_{\mathrm{1,2}}=1.97$ and $\:{n}_{\mathrm{2,2}}=2$ and bottom cell $\:{n}_{\mathrm{1,3}}=1$ and $\:{n}_{\mathrm{2,3}}=2$ that is consistent with the near-ideal behavior of the Ge bottom sub-cell confirmed experimentally by Roensch et al.^18^. These values are consistent with the parameterization of Catelani et al.^7^ and Anaty et al.^6^ adopted in the present model.

The series connection imposes current matching: the terminal current equals the minimum sub-cell photocurrent, and the terminal voltage is the algebraic sum of all sub-cell voltages:

9 $$\:{I}_{Total}=\mathrm{m}\mathrm{i}\mathrm{n}({I}_{1}\:,\:{I}_{2}\:,\:{I}_{3})$$

10 $$\:{V}_{Total}={V}_{1}+\:{V}_{2}+\:{V}_{3}$$

The sub-cell open-circuit voltage dependence on temperature and geometric concentration ratio follows the analytical model of Helmers et al.^16^:

11 $$\:{V}_{OC,\boldsymbol{j}}={V}_{OC,\boldsymbol{j}}^{Ref}-\:{\beta\:}_{Voc,\boldsymbol{j}}\left({T}_{c}-{T}_{ref}\right)+\:\frac{\stackrel{-}{{n}_{\boldsymbol{j}}}\:\mathrm{k}\:{T}_{c}}{q}\:\mathrm{ln}\left({CR}_{g}\right)$$

where $\:{\beta\:}_{Voc,\boldsymbol{j}}$ is the measured subcell-specific V_OC_ temperature coefficient (mV K^− 1^): -2.8 for GaInP, -2.4 for GaInAs, and − 2.1 for Ge^16^. Bandgap temperature dependence for each sub-cell uses the Varshni equation^26^, with material parameters for GaInP (E_g,0 K_ = 1.90 eV), GaInAs (1.49 eV), and Ge (0.75 eV)^15^.

### Receiver thermal model

The energy balance on the receiver aperture couples optical input, electrical generation, thermal extraction, and thermal loss:

12 $$\:{S}_{absorbed}={P}_{electric}+\:{Q}_{th,useful}+\:{Q}_{loss}$$

Where $\:{S}_{absorbed}$ is the absorbed solar power, $\:{P}_{electric}$ is electric power generated, $\:{Q}_{th,useful}$ is the recoverable thermal energy and $\:\:{Q}_{loss}$ is thermal losses energy.

The recoverable thermal power extracted from the coolant fluid is:

13 $$\:{Q}_{th,useful}=\:\dot{\:{m}_{f}}\:\cdot \:\:{C}_{p}\:\cdot \:\:\left({T}_{f,out}-{T}_{f,in}\right)$$

Receiver thermal loss to the environment combines convective and radiative contributions and is modelled as:

14 $$\:{Q}_{loss}=\:{U}_{L}\:\cdot \:\:{A}_{rec}\:\cdot \:\left({T}_{c}-{T}_{amb}\right)$$

where U_L_ is the overall heat loss coefficient (W m^− 2^ K^− 1^), A_rec_ is the receiver outer surface area, $\:{T}_{c}$ is the mean cell or tube temperature, and $\:{T}_{amb}$ is the ambient temperature.

The coolant outlet temperature $\:{T}_{f,out}$_t_ is determined iteratively from the coupled heat transfer and PV electrical model at each simulation timestep. The overall heat loss coefficient U_L_ is computed dynamically at each hourly timestep within the SIMSCAPE thermal domain, as a function of the instantaneous wind speed, ambient temperature, and receiver surface temperature. This dynamic treatment ensures that seasonal variations in wind speed and ambient conditions are fully captured in the thermal loss calculation throughout the year.

### PEM electrolyzer model

The PEM electrolyzer model follows the comprehensive electrochemical-thermal framework developed and validated against experimental data by Bessarabov et al.^27^ and Aouali et al.^8^ and implemented in the MATLAB/SIMSCAPE environment. The electrolyzer cell voltage is expressed as the sum of the Nernst reversible equilibrium potential and three overpotential contributions:

15 $$\:{V}_{cell}={E}_{rev}+{\eta\:}_{act}+{\eta\:}_{ohm}+{\eta\:}_{conc}$$

Where $\:{V}_{cell}$ is the electrolyzer cell voltage, $\:{E}_{rev}$ is the reversible potential, $\:{\eta\:}_{act}$ is the activation overpotential, $\:{\eta\:}_{ohm}$ is the ohmic overpotential and $\:{\eta\:}_{conc}$ is the concentration overpotential.

The temperature reversible Nernst potential accounts for operating temperature $\:{T}_{el}$ and product gas partial pressures^27^:

16 $$\:{E}_{\mathrm{r}\mathrm{e}\mathrm{v}}={\mathrm{E}}_{th}^{0}+\frac{{R}_{u}{T}_{el}}{2F}\mathrm{l}\mathrm{n}\left(\frac{{p}_{{\mathrm{H}}_{2}}\cdot\:{p}_{{\mathrm{O}}_{2}}^{1/2}}{{a}_{{\mathrm{H}}_{2}\mathrm{O}}}\right)$$

where, $\:{\mathrm{E}}_{th}^{0}$ is standard reversible potential (1.229 V at 25 °C), $\:{R}_{u}$ is universal gas constant (8.314 J/(mol⋅K)), $\:{T}_{el}$ is cell operating temperature (kelvin), *\:F* is Faraday’s constant (96485 C/mol), $\:{p}_{{\mathrm{H}}_{2}},{p}_{{\mathrm{O}}_{2}}$ are partial pressures of hydrogen and oxygen (bar), $\:{a}_{{\mathrm{H}}_{2}\mathrm{O}}$ is activity of liquid water (≈ 1 for pure water).

Activation overpotential at the anode (a) and cathode (c) is modelled by:

17 $$\:{\eta\:}_{act}={\eta\:}_{activation}^{anode}+\left|{\eta\:}_{activation}^{cathode}\right|$$

where, the anode or cathode activation overpotential is calculated as^28^:

18 $$\:{\eta\:}_{act,x}=\frac{{R}_{u}{T}_{el}}{{\alpha\:}_{x}{z}_{x}F}\cdot \:\mathrm{l}\mathrm{n}\left(\frac{i}{{i}_{0,x}}\right)$$

where, *\:x* stands for anode or cathode, $\:{\alpha\:}_{x}$ is the anode ($\:{\alpha\:}_{a}=0.51$) or cathode ($\:{\alpha\:}_{c}=0.627$) charge transfer coefficient, $\:{z}_{x}$ is the stoichiometric coefficient of electrons for the anode ($\:{z}_{a}=3$) or cathode ($\:{z}_{c}=-1$) reaction, *\:i* is the operating current density (A/cm²), $\:{i}_{0,x}$ is the exchange current density at the anode or cathode (A/cm²).

The anode or cathode exchange current density ($\:{i}_{0,x}$) is a critical parameter that is highly dependent on temperature and catalyst properties. It is modeled using an Arrhenius-type expression^27^:

19 $$\:{i}_{0,x}={i}_{0,\mathrm{\:ref\:},x}\cdot\:{\gamma\:}_{m,x}\cdot\:{C}_{{H}_{2}O,\mathrm{\:ratio\:}}\cdot\:\mathrm{e}\mathrm{x}\mathrm{p}\left(-\frac{{E}_{excit,x}}{{R}_{u}}\left(\frac{1}{{T}_{el}}-\frac{1}{{T}_{\mathrm{ref\:}}}\right)\right)$$

where, $\:{i}_{0,\mathrm{\:ref\:},x}$ is reference exchange current density for the anode ($\:{i}_{0,\mathrm{\:ref\:},a}=1{\times\:10}^{-7}$) or cathode ($\:{i}_{0,\mathrm{\:ref\:},c}=1{\times\:10}^{-3}$), $\:{C}_{{H}_{2}O,\mathrm{\:ratio\:}}$ is the ratio of water concentration to reference concentration anode ($\:{C}_{{H}_{2}O,\mathrm{\:ratio\:}}=0.22$), $\:{E}_{excit,x}$ is the excitation energy for the anode ($\:{E}_{excit,a}=76{\times\:10}^{3}$) or cathode ($\:{E}_{excit,c}=4.3{\times\:10}^{3}$) reaction (J/mol), $\:{T}_{\mathrm{ref\:}}$ is the reference temperature for the excitation parameters and equals to 353 K (K).

And the catalyst surface area factor ($\:{\gamma\:}_{m,x}$) for the platinum catalyst is calculated as^27,29^:

20 $$\:{\gamma\:}_{m,x}={\phi\:}_{x}\cdot\:{m}_{cat,x}\cdot\:\left(\frac{6}{{\rho\:}_{x}\cdot\:{d}_{cat,x}}\right)$$

where, $\:{\phi\:}_{x}$ is the fraction of active metal catalyst for anode or cathode anode ($\:{\phi\:}_{x}=0.75$), $\:{m}_{cat,x}$ is the catalyst loading at the anode ($\:{m}_{cat,a}=1{\times\:10}^{-3}$) or cathode ($\:{m}_{cat,c}=0.3{\times\:10}^{-3}$) (g/cm²), $\:{\rho\:}_{x}$ is the density of the anode which is platinum catalyst or of the cathode which is iridium catalyst (g/cm³), $\:{d}_{cat,x}$ is the catalyst crystallite diameter for anode ($\:{d}_{cat,a}=2.9{\times\:10}^{-7}$) or cathode ($\:{d}_{cat,a}=2.7{\times\:10}^{-7}$) (cm).

The ohmic overpotential accounts for the voltage losses due to resistance to the flow of charge within the electrolyzer cell

21 $$\:{\eta\:}_{act}={\eta\:}_{Ionic}+{\eta\:}_{electronic}$$

The ionic overpotential $\:{\eta\:}_{Ionic}$ arises from the resistance of the membrane to proton transport. The electronic overpotential, which accounts for the resistance in the solid components of the cell, is typically much smaller than the ionic overpotential and it is assumed to be a small, fixed fraction of the ionic overpotential equals to 1% of ionic overpotential. Ionic overpotential is calculated as:

22 $$\:{\eta\:}_{Ionic}=i\:\cdot \:\:\frac{{\mathrm{t}}_{memb}^{total}}{{\sigma\:}_{memb}}$$

where, *\:i* is the operating current density (A/cm^2^), $\:{\mathrm{t}}_{memb}^{total}$ is the total thickness of the membrane and gas diffusion layers (cm), $\:{\sigma\:}_{memb}$ is the ionic conductivity of the membrane (S/cm).

The membrane conductivity ($\:{\sigma\:}_{memb}$) is a sensitive parameter that depends on the membrane’s hydration level and operating temperature^29^

23 $$\:{\sigma\:}_{\mathrm{memb\:}}={\left({\epsilon}_{\mathrm{mem\:}}-{\epsilon}_{0,\mathrm{\:mem\:}}\right)}^{1.5}\cdot\:\left(\frac{\beta\:}{18{\lambda\:}_{\mathrm{sorbed\:}}}\right)\cdot\:\left(\frac{394.8}{1+{\delta\:}_{\mathrm{memb\:}}}\right)\cdot\:\mathrm{e}\mathrm{x}\mathrm{p}\left(-\frac{{E}_{\mathrm{viscosity\:},{H}_{2}O}}{{R}_{u}}\left(\frac{1}{{T}_{el}}-\frac{1}{298}\right)\right)$$

where, $\:{\delta\:}_{\mathrm{memb\:}}$ is a dimensionless fitting constant for the conductivity model ($\:{\delta\:}_{\mathrm{memb\:}}=1.65$), $\:{E}_{\mathrm{viscosity\:},{H}_{2}O}$ is the activation energy related to water viscosity ($\:{E}_{\mathrm{viscosity\:},{H}_{2}O}=14000$) (J/mol), $\:{\epsilon}_{\mathrm{mem\:}}$, is the membrane porosity which is a function of the number of sorbed water molecules per $\:{\lambda\:}_{\mathrm{sorbed\:}}$ acid site ($\:{\lambda\:}_{\mathrm{sorbed\:}}=20\:,\:{\lambda\:}_{0,\mathrm{sorbed\:}}=1.8$) and the ratio of membrane molar volume to water (*r* = 30)^27,30^:

24 $$\:{\epsilon}_{\mathrm{mem\:}}=\frac{{\lambda\:}_{\mathrm{sorbed\:}}}{{\lambda\:}_{\mathrm{sorbed\:}}+r}\:\:,\:\:{\epsilon}_{0,\mathrm{\:mem\:}}=\frac{{\lambda\:}_{0,\mathrm{sorbed\:}}}{{\lambda\:}_{0,\mathrm{sorbed\:}}+r}$$

$\:\beta\:$ is the degree of acid site dissociation, given by:

25 $$\:\beta\:=\frac{\left({\lambda\:}_{\mathrm{sorbed\:}}+1\right)-\sqrt{{\left({\lambda\:}_{\mathrm{sorbed\:}}+1\right)}^{2}-4{\lambda\:}_{\mathrm{sorbed\:}}\left(1-\frac{1}{{K}_{\mathrm{acid\:}}}\right)}}{2-\frac{2}{{K}_{\mathrm{acid\:}}}}$$

And $\:{K}_{\mathrm{acid\:}}$ is the dissociation equilibrium constant^27,30^:

26 $$\:{K}_{\mathrm{acid\:}}=\mathrm{e}\mathrm{x}\mathrm{p}\left(-\frac{52300}{{R}_{u}}\left(\frac{1}{{T}_{el}}-\frac{1}{298}\right)\right)$$

Concentration total overpotential at high current densities is calculated as:

27 $$\:{\eta\:}_{conc}={\eta\:}_{conc}^{anode}+\left|{\eta\:}_{conc}^{cathode}\right|$$

The concentration overpotential at the anode or cathode is calculated as^27^:

28 $$\:{\eta\:}_{conc,x}=\frac{{R}_{u}{T}_{el}}{{\alpha\:}_{x}{z}_{x}F}\cdot \:\mathrm{l}\mathrm{n}\left(\frac{{i}_{limit}}{{i}_{limit}-i}\right)$$

where, $\:{i}_{limit}$ is the limiting current density (A/cm^2^), a key parameter representing the maximum current that can be sustained before mass transport limitations become critical ($\:{i}_{limit}=4\:\mathrm{A}/\mathrm{c}\mathrm{m}^{2}$).

### Multi-effect evaporation desalination model

The MEE desalination unit is modelled as a forward-feed system with N_eff_ = 6 serially connected effects operating at successively decreasing temperatures driven by the receiver thermal output. The MEE desalination unit was implemented as a physics-based dynamic model within the MATLAB/SIMSCAPE environment, constructed using standard blocks from the SIMSCAPE Fluids Two-Phase Fluid library. This approach provides a first-principles representation of the core thermodynamic phenomena of evaporation and condensation which govern MEE plant performance, rather than relying on simplified steady-state performance correlations. The primary output of the model at each simulation timestep is the instantaneous freshwater production rate, computed dynamically as a function of the thermal energy supplied by the solar field. The heat transfer within each effect is computed using the SIMSCAPE Two-Phase Fluid library’s internal two-phase convection model, which accounts for boiling and condensation phenomena beyond the single-phase Nusselt correlation of the pipe block. The complete MEE SIMSCAPE model was validated against the performance data of El-Dessouky and Ettouney^31^ before its integration into the polygeneration simulation platform, confirming the reliability of the mass and energy balance calculations across all six effects.

**Fig. 3:**
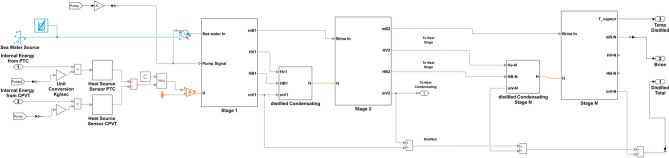
Full model structure for MEE in SIMSCAPE.

The model was built with a modular architecture in which each effect of the MEE train was developed as an independent SIMSCAPE subsystem. The six subsystems were then connected in series to form the complete multi-effect train, mirroring the physical construction of a real forward-feed MEE plant and allowing straightforward reconfiguration for a different number of effects. Within each effect, four physical processes are represented by dedicated SIMSCAPE sub-blocks: (i) simultaneous evaporation and condensation in the inter-effect heat exchanger, modelled using a pipe block with two-phase fluid domain; (ii) phase separation, implemented via a saturation properties sensor coupled with a vapor quality sensor to determine the thermodynamic state and mass fraction of the produced vapor; (iii) inter-stage thermal coupling, in which the brine and distillate thermodynamic properties computed at the outlet of each effect are used to calculate the motive heat input available to the subsequent effect; and (iv) distillate collection, in which the condensate produced in the heat exchanger of each effect is accumulated and summed to yield the total plant freshwater output as seen in Fig. 3.

### Technoeconomic analysis

The techno-economic analysis translates the annual simulation outputs like hydrogen mass, freshwater, and exported electricity into standardized economic metrics that allow direct comparison of different system configurations. The methodology is based on a discounted cash flow analysis over the full plant operational lifetime n, accounting for all capital expenditure, operational costs, replacement costs, and co-product revenues.

The total capital expenditure (CAPEX) represents the upfront investment required to construct the integrated system, encompassing the solar field, concentrating receiver, PEM electrolyzer stack, MEE desalination unit, and balance-of-plant costs including installation works. Annual operational expenditure (OPEX) comprises a fixed component as scheduled maintenance, insurance, and labor, assumed as a fixed percentage of CAPEX and a variable component covering consumables and periodic component replacements such as in electrolyzer stack.

The present value of any uniform annual quantity A over the plant lifetime is calculated as:

29 $$\:\mathrm{N}\mathrm{P}\mathrm{V}=A\times\:\left[\frac{1-(1+i{)}^{-n}}{i}\right]$$

where *\:i* is the real discount rate and *\:n* the plant lifetime in years. This formulation is applied consistently to all recurring annual cost and revenue streams.

The primary economic output is the Levelized Cost of Hydrogen (LCOH), representing the average cost of producing one kilogram of hydrogen over the plant lifetime after crediting all co-product revenues:

30 $$\:LCOH({\$}/{\rm kg})=\frac{\mathrm{\:NPV(Total\:Plant\:Costs)\:}-\mathrm{\:NPV(}\mathrm{\:Product\:}\mathrm{Revenue)\:}}{\mathrm{\:NPV(Hydrogen\:Production)\:}}$$

where: NPV (Total Plant Costs) = Total CAPEX + NPV (All Replacement Costs) + NPV(Total Annual OPEX) ; NPV (Product Revenue) = NPV(Annual Co-Product Production × Market Price of Co-Product); NPV (Hydrogen Production) = NPV(Annual Hydrogen Production in kg).

In polygeneration-I configurations, the co-product revenue term includes freshwater sales. In polygeneration-II configurations, it includes exported electricity revenues. The key financial and cost parameters adopted in this analysis are summarized in Table 3.

Finally, the polygeneration system efficiency on an HHV basis is defined as:

31 $$\:{\eta\:}_{sys}^{HHV}=\frac{\text{}{m}_{H2}^{annaul}\text{}\cdot \mathrm{\:HHV}-\text{}{\mathrm{Energy}}_{export\:}^{annual}\text{}}{\text{}{Solar\:Energy}^{annual}\text{}}$$

## Simulation methodology and environment

### Simulation platform

**Fig. 4:**
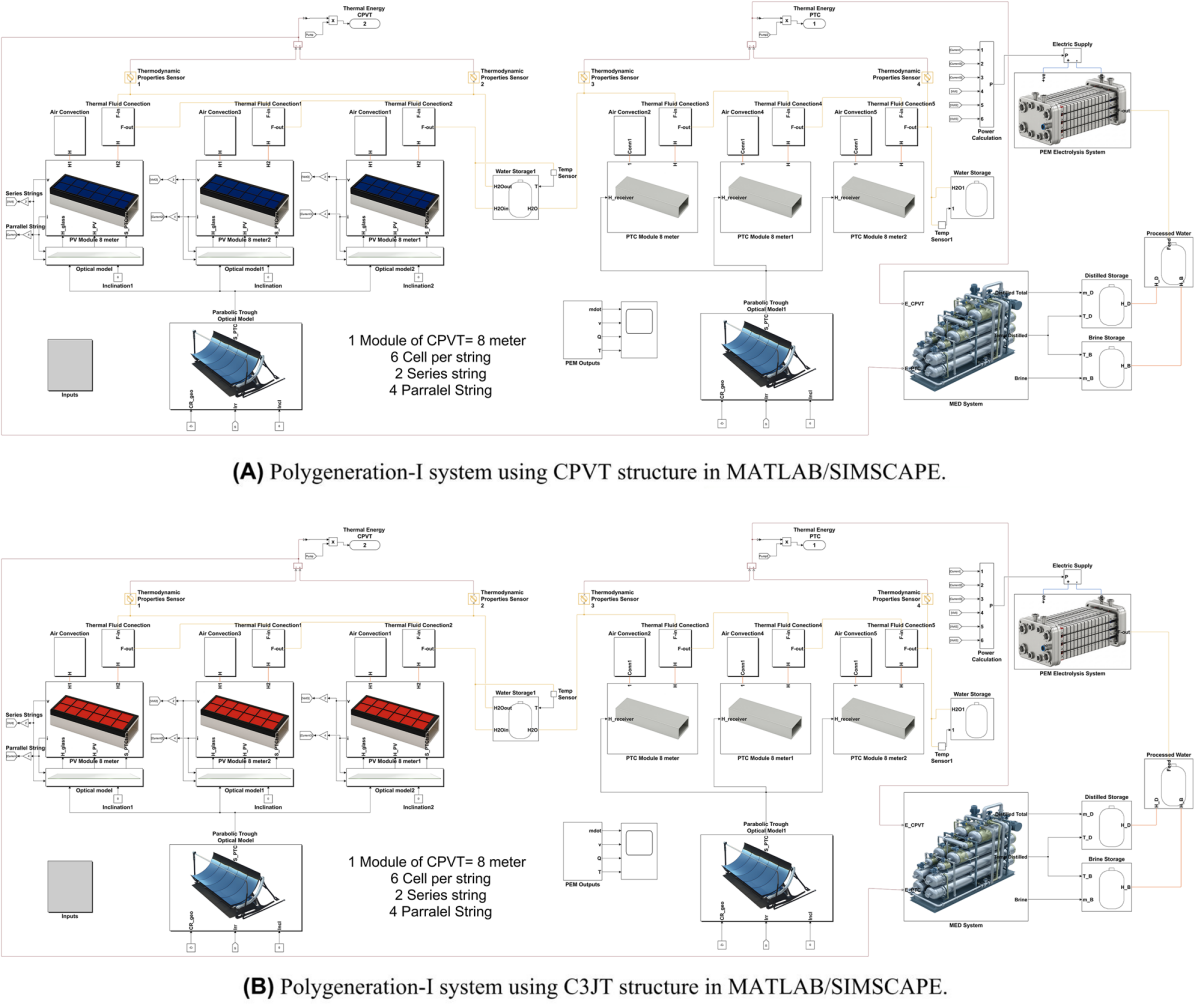
Integrated system implementation for polygeneration-I in MATLAB/SIMSCAPE.

**Fig. 5:**
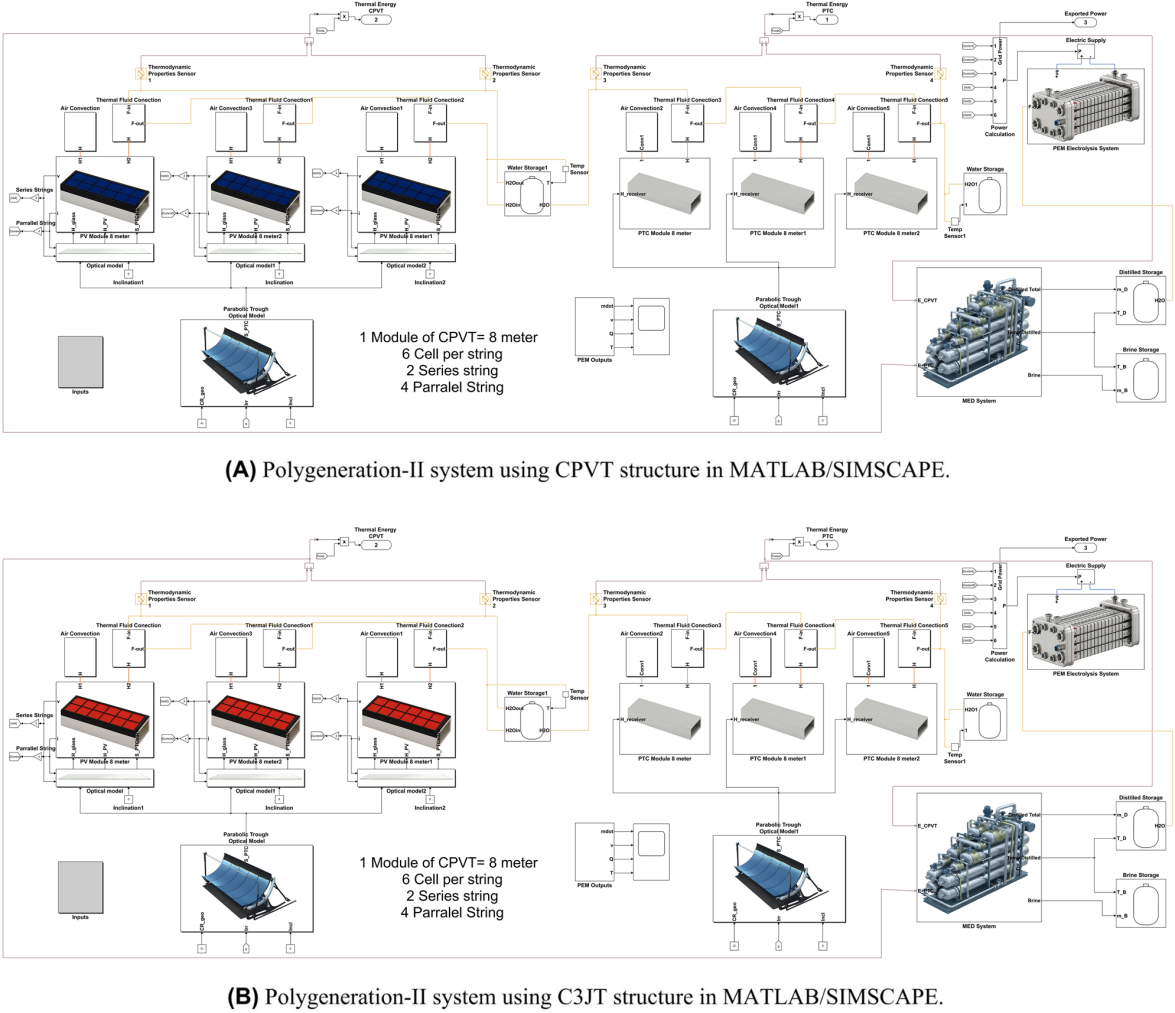
Integrated system implementation for polygeneration-II in MATLAB/SIMSCAPE.

All simulations were performed in MATLAB using the SIMSCAPE physical modelling environment as shown in Figs. 4 and 5. The integrated polygeneration system that contains the concentrating solar collector, CPVT or C3JT receiver, PEM electrolyzer, and MEE desalination unit, was implemented as a single coupled SIMSCAPE model and executed as a continuous hour-by-hour transient simulation over a full representative meteorological year of 8,760 h. This approach captures the dynamic coupling between all subsystems at each hourly timestep, including the seasonal variation of solar input, the temperature-dependent behavior of the PV receivers and PEM electrolyzer, and the instantaneous thermal energy available to drive the MEE desalination unit. Each subsystem component was individually validated against published experimental data from the literature prior to integration into the full system model. These validations of individual system components contribute to high accuracy of the integrated system level performance. The simulation employs a fixed timestep of 3,600 s, consistent with the hourly resolution of the METEONORM meteorological input data. METEONORM generates hourly time series from long-term measured ground-based and satellite records. The SIMSCAPE solver uses the ode23t stiff differential equation solver with a relative tolerance of 1 × 10^− 3^ and an absolute tolerance of 1 × 10^− 6^. Convergence at each timestep is confirmed when the algebraic loop residual falls below 1 × 10^− 6^. The coupled thermal, electrical, electrochemical, and fluid domains are solved simultaneously within the SIMSCAPE physical network, eliminating the sequential operator-splitting error propagation that would arise in a weakly coupled co-simulation framework. Simulation parameters for different polygeneration subsystems in the selected locations in Egypt are included in Table 2.

**Table 2:** Simulation parameters for polygeneration systems in different locations in Egypt.

	Site		
	Hurghada	Suez	Port Said
Location	27.2188°N/ 33.6559°E	29.7280°N/ 32.3431°E	31.1023°N/ 32.2119°E
Seawater salinity (g/kg)	42.5	44.0	38.5

Parameter	Unit	Value/description	
**Solar field – general**			
Total collector aperture area	m²	240	
**Solar concentrator**			
Concentrator type		Parabolic Trough Collector (PTC)	
Geometric concentration ratio	-	32x	
**Heat transfer fluid (HTF)**			
HTF type	-	Water	
HTF mass flow rate in CPVT	kg/s	0.383	
HTF mass flow rate in PTC	kg/s	0.5	
**PEM electrolyzer**			
Number of cells	-	50	
Operating pressure	Bar	1	
**MEE desalination**			
Mass flow rate	kg/s	0.203	
Number of effects	-	6	
Heat transfer area per effect	m^2^	1.6	
Top brine temperature	°C	72	
Last effect temperature	°C	40	

### Economic input parameters

The capital expenditure (CAPEX) values for each system component were sourced from recent peer-reviewed literature and technical reports for MENA-region solar hydrogen projects. Annual operational expenditure (OPEX) was assumed as a fixed percentage of total CAPEX, covering scheduled maintenance, insurance, and labor costs. The key financial and cost parameters adopted in this analysis are summarized in Table 3.

**Table 3:** Economic parameters for LCOH and polygeneration analyses.

Economics			
Parameter	Unit	Value	Reference
**Financial parameters**			
Project lifetime	Years	25	-
Discount rate	%	8	-
**Capital costs (CAPEX)**			
Parabolic trough concentrating system	$\:{\$}/{\rm m}^{2}$	120	^32^
PV cell	$\:{\$}/{\rm m}^{2}$	150	^33^
Triple-junction (C3JT) system	$\:{\$}/{\rm m}^{2}$	450	
PEM electrolyzer	$\:{\$}/{\rm k}\mathrm{W}$	500	^34^
H_2_ compressor cost	$\:{\$}/{\rm kg}_{\rm H2}$	2.49	^35^
H_2_ & HTF storage vessel cost	$\:{\$}/{\rm kg}_{\rm H2}$	516	
Indirect costs	%CAPEX	10	-
**Operational costs (OPEX)**			
CPVT maintenance rate	%CAPEX	1	-
PEM maintenance rate	%CAPEX	1	-
PEM water purification rate	$\:{\$}/{\rm kg}_{\rm H2}$	1	-
H_2_ compressor & storage maintenance rate	%CAPEX	10	-
Insurance & consumables rate	%CAPEX	1	-
PEM stack replacement cost	$\:{\$}/\mathrm{k}\mathrm{W}$	264	^34^
Electricity price (variable tariff)	$\:{\$}/\mathrm{k}\mathrm{W}\mathrm{h}$	0.1	-
Freshwater market price	$\:{\$}/{\rm m}^{3}$	1	^11^

### Sub-model validation

**Fig. 6:**
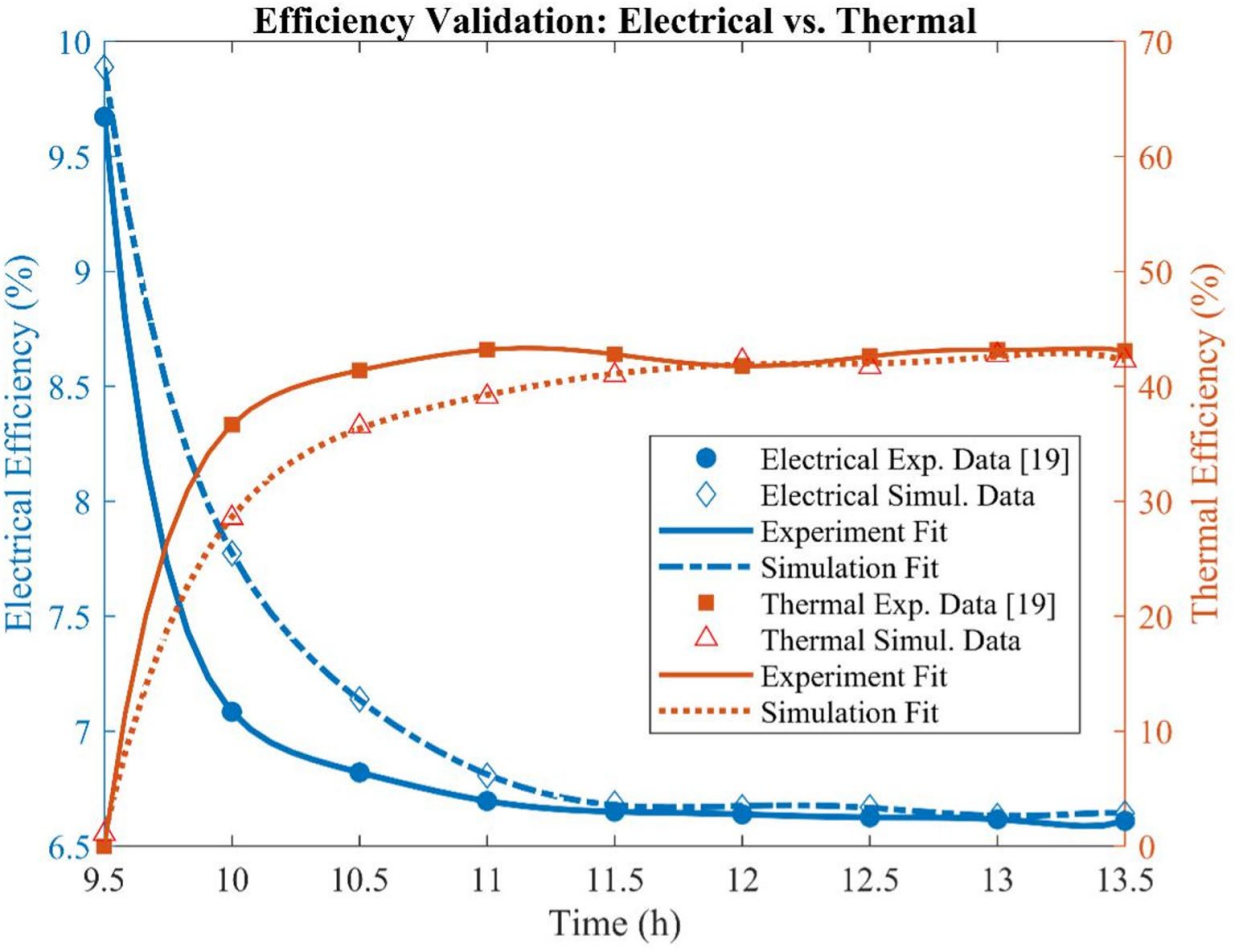
Efficiency validation for CPVT model.

**Fig. 7:**
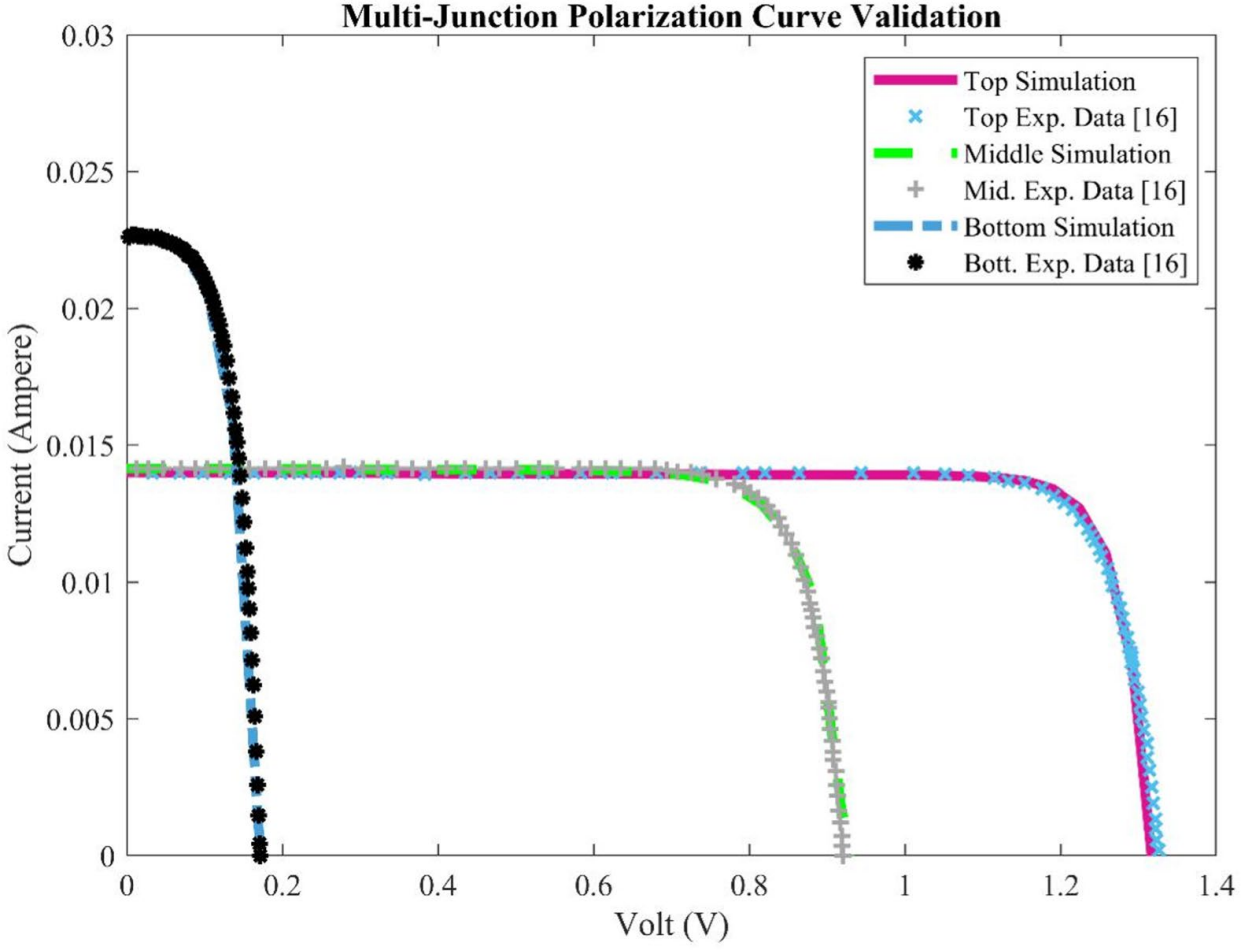
Polarization curve validation for triple junction model.

**Fig. 8:**
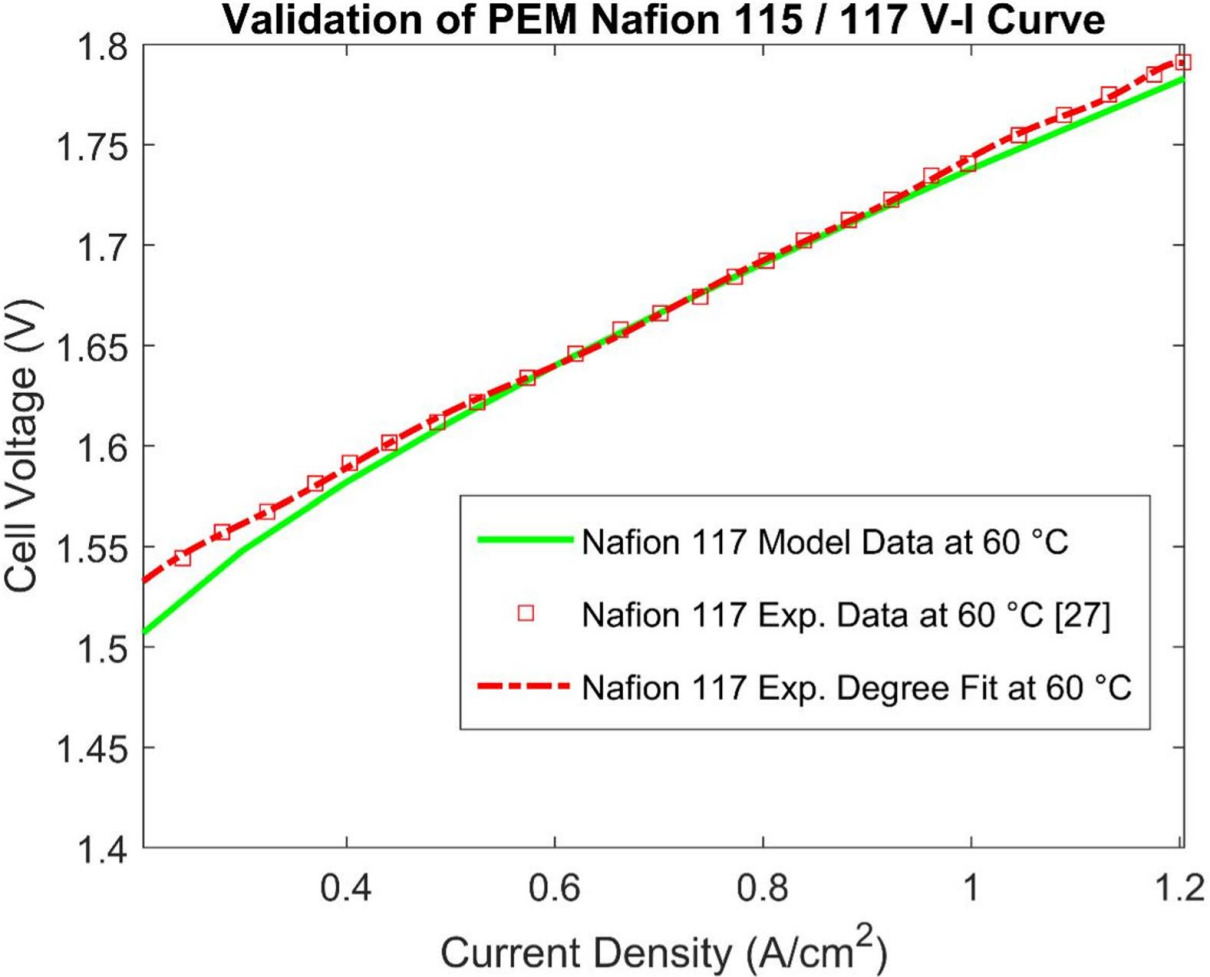
Polarization curve validation for PEM electrolyzer model.

The models of CPVT receiver, C3JT receiver, PEM electrolyzer, and MEE desalination units were developed in MATLAB/SIMSCAPE and validated against experimental data. A summary of the validation metrics is given in Table 4. The CPVT receiver model was validated against the experimental measurements of Riahi et al.^19^ shown in Fig. 6, the C3JT triple-junction cell model against the datasets of Helmers et al.^16^ shown in Fig. 7, the PEM electrolyzer model against the polarization curve data Bessarabov et al.^27^ shown in Fig. 8, and the MEE desalination block against the performance data of El-Dessouky and Ettouney^31^.

**Table 4:** Summary of sub‑models validation metrics.

Component	Parameter	MAPE	RMSE
CPVT	Electrical efficiency	2.3%	0.267%
CPVT	Thermal efficiency	6.74%	3.535%
C3JT	Polarization curve	7.58% (top)	0.0011 A
		2.74% (middle)	0.0004 A
		5.72% (bottom)	0.0013 A
PEM electrolyzer	Polarization curve	1.160%	0.030 V
MEE	Distillate flow	0.038%	-

## Results and discussions

The results of this study are presented in three progressive layers. First, the solar resource characteristics and annual electrical performance of both receiver technologies at the three Egyptian locations are established as the common reference baseline. Second, the performance of polygeneration-I configurations (Cases A and B) is analyzed in terms of hydrogen yield, thermal output, freshwater production, and system efficiency. Third, polygeneration-II configurations (Cases A and B) are examined, with a focus on electricity export, the consequent reduction in hydrogen production, and the effect on overall efficiency. A comprehensive techno-economic analysis concludes the section, examining the LCOH across all cases and benchmarking results against published literature.

### Solar resource and annual electrical output

Annual solar energy at the three Egyptian Sea coast locations was simulated on hourly basis. The key meteorological parameters governing system performance at each location are summarized in Table 5. Hurghada records the highest annual DNI at 1,872.4 kWh m^− 2^ yr^− 1^, followed by Port Said at 1,798.8 kWh m^− 2^ yr^− 1^ and Suez at 1,752.9 kWh m^− 2^ yr^− 1^. Although the ranking appears straightforward, the differences are non-trivial: Hurghada’s DNI advantage over Suez amounts to 119.5 kWh m^− 2^ yr^− 1^ (6.8%), a gap large enough to produce meaningful differences in all downstream outputs. However, DNI alone does not determine system performance as Table 5 shows that the three locations also differ in annual sunshine hours, mean ambient temperature, and mean wind speed, all of which influence the thermal losses, cell operating temperatures, and effective operating hours of the integrated system. These differences become particularly important when interpreting efficiency, where locations with lower DNI do not always produce lower efficiency. The hourly DNI profile follows a strong seasonal envelope, with peak hourly intensities reaching approximately 900 W m^− 2^ during summer months and declining to near zero during winter nights, a pattern that directly governs the dynamics of all system outputs discussed in subsequent sections.

**Table 5:** Meteorological parameters from simulations at each location.

Location	Annual DNI (kWh/m^2^/yr)	Sunshine hours (hr/yr)	Mean ambient temp (°C)	Mean wind speed (m/s)
Hurghada	1,872.4	3,986	25.78	5.958
Suez	1,752.9	3,966	23.48	3.114
Port Said	1,798.8	3,913	21.83	3.643

**Fig. 9:**
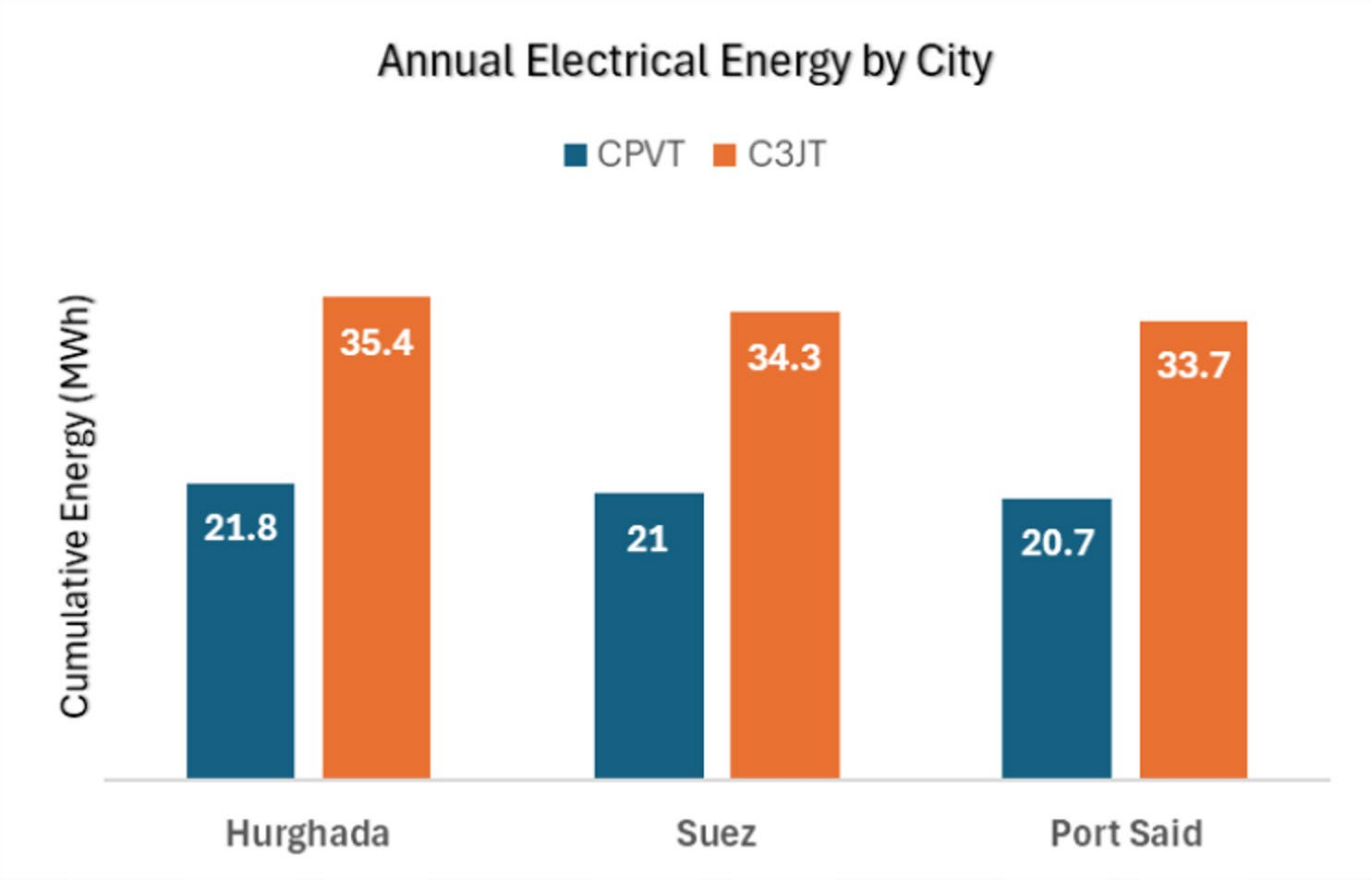
Comparative annual electricity production for different technologies.

The annual electrical output profile for both receiver technologies was obtained from the hourly dynamic simulation and shows the same seasonal envelope as the DNI resource. Figure 9 shows the cumulative electrical energy which represents a consistent advantage of approximately 62–63% for C3JT over the CPVT at all three sites that proof the higher solar conversion efficiency of triple junction cells.

**Fig. 10:**
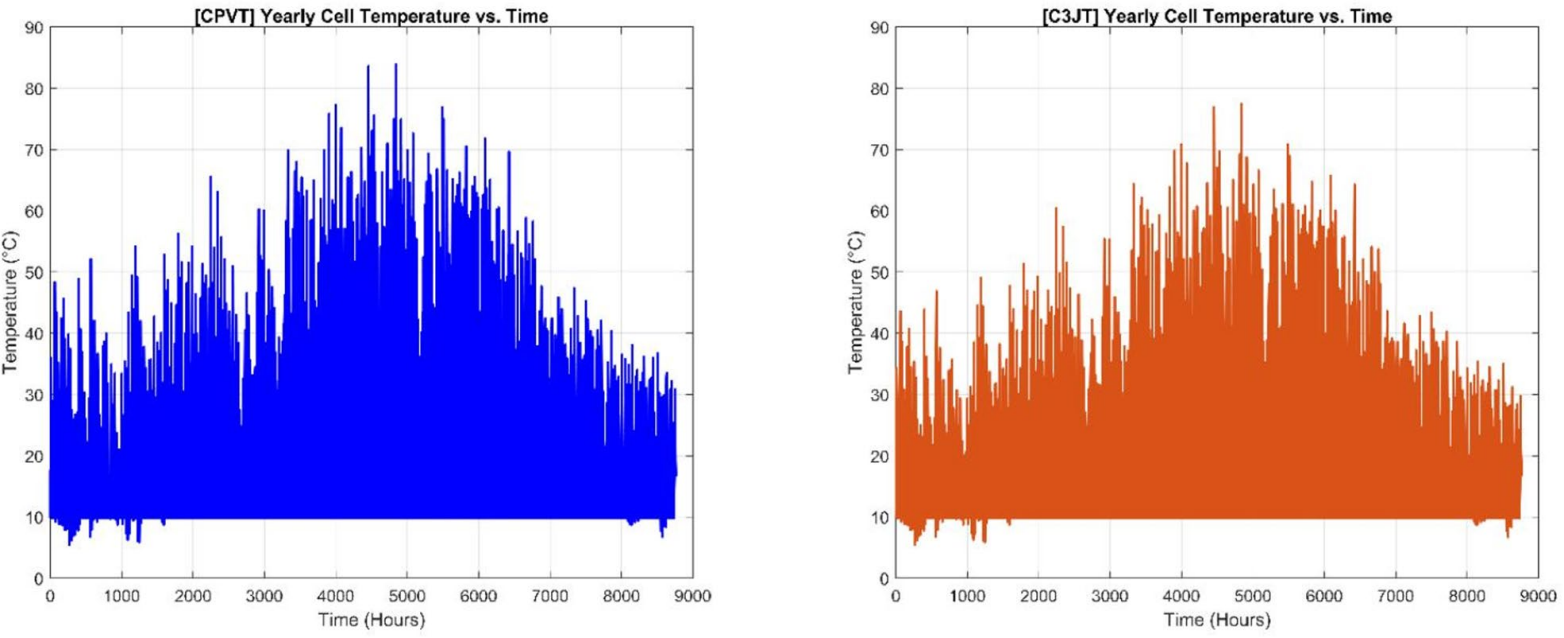
Annual cell temperature analysis for different technologies.

The annual cell temperature profiles for Hurghada are shown in Fig. 10 reveal a physically important distinction between the two receiver types. The CPVT cells operate at average temperatures approximately 5 °C higher than the C3JT cells under identical solar input conditions. This difference is due to the lower photoelectric conversion efficiency of the CPVT cell which means a larger fraction of absorbed solar energy is dissipated as heat. The superior performance of the C3JT receiver is driven by three interconnected mechanisms. Spectrally, the triple-junction architecture partitions the solar spectrum among three sub-cells with complementary bandgaps minimizing thermalization and sub-bandgap losses that limit single-junction silicon cells. Electrically, the series connection sums three sub-cell voltages yielding in a higher conversion efficiency than for CPVT. Thermally, higher electrical efficiency reduces waste heat, which further improves efficiency.

### Polygeneration-I: hydrogen and fresh water

#### Annual hydrogen production

The annual hydrogen production is presented in Table 6 for polygeneration-I, Cases A and Cases B at all three locations. The C3JT receiver represents improvements of 57.2%, 57.6%, and 57.1% over the corresponding CPVT values. The consistency of this percentage advantage across all three locations, which differ in DNI, confirms that the performance ratio between the two technologies is governed primarily by receiver characteristics rather than by location-specific solar resource variations.

**Table 6:** Annual hydrogen and distilled production for Cases I-A and I-B at different locations.

Location	H₂ I-A (kg/yr)	H₂ I-B (kg/yr)	Distilled I-A (kg/yr)	Distilled I-B (kg/yr)
Hurghada	521.2	819.3	660.0	466.9
Suez	503.0	793.1	581.1	399.5
Port Said	496.4	779.6	622.6	442.5

**Fig. 11:**
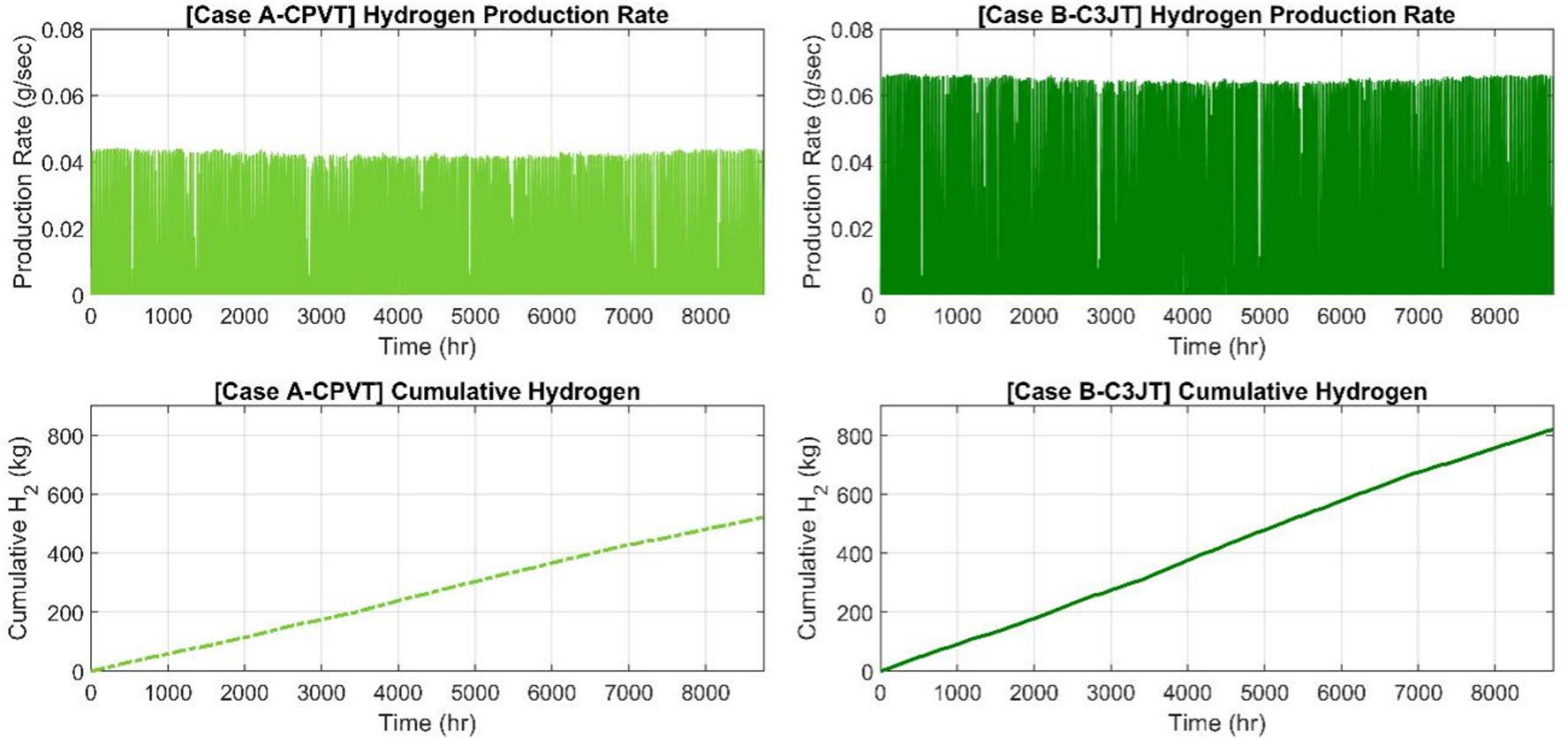
Hourly and cumulative annual hydrogen production for Cases I-A and I-B in Hurghada.

The hourly hydrogen production rate profiles shown in Fig. 11, reveal the dynamic behavior of both configurations across the 8,760-hour simulation year. The instantaneous production rate (g/s) follows the direct normal irradiance seasonal envelope closely and falls to zero during all non-daylight hours. This on/off daily cycling pattern, repeated across 365 days, produces the annual totals reported in Table 6. The C3JT profiles are consistently shifted upward relative to the CPVT profiles throughout the entire year confirming that the technology advantage is uniform across all solar conditions.

#### Thermal energy and freshwater production

**Fig. 12:**
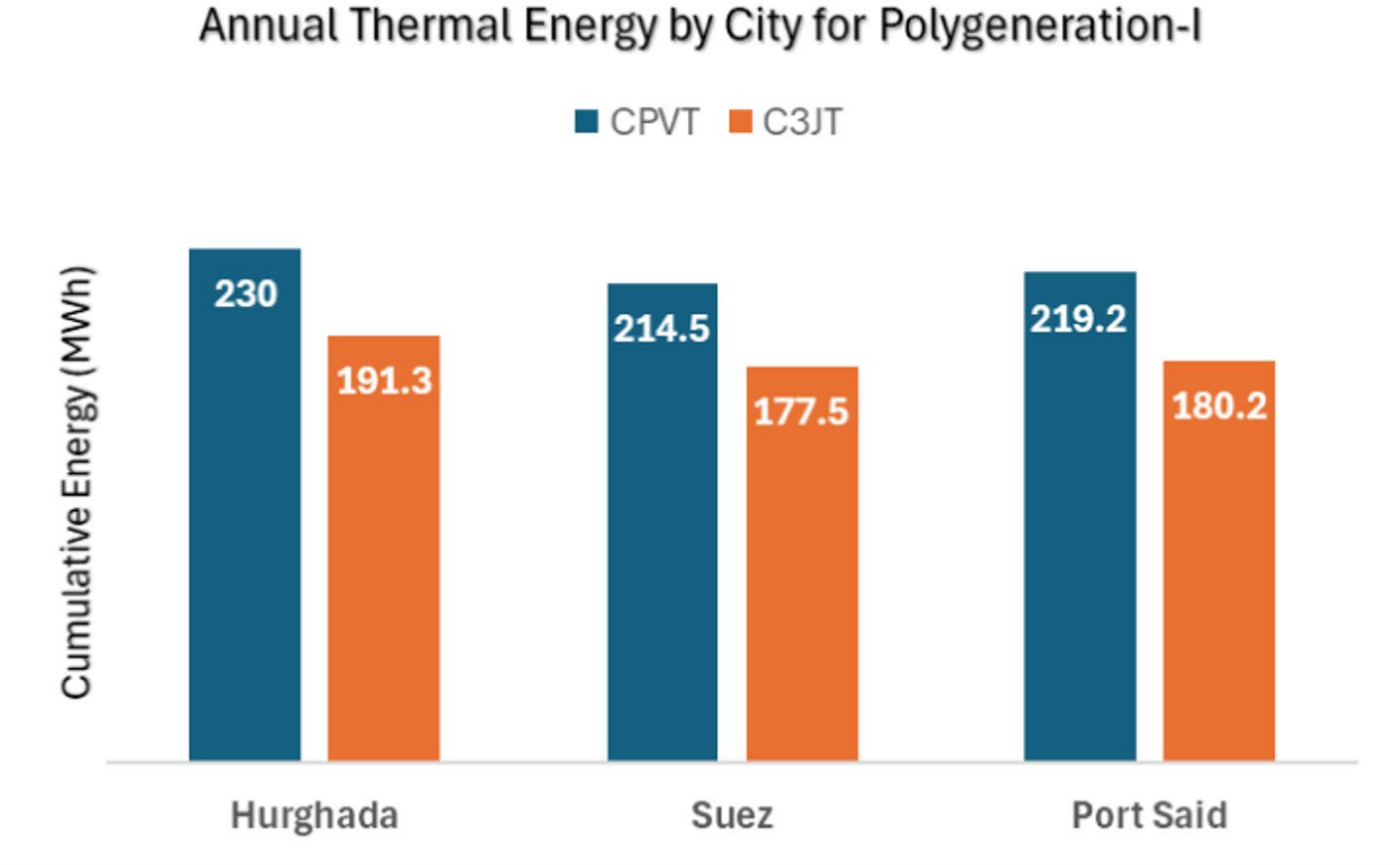
Cumulative annual thermal production for Cases I-A and I-B at different locations.

**Fig. 13:**
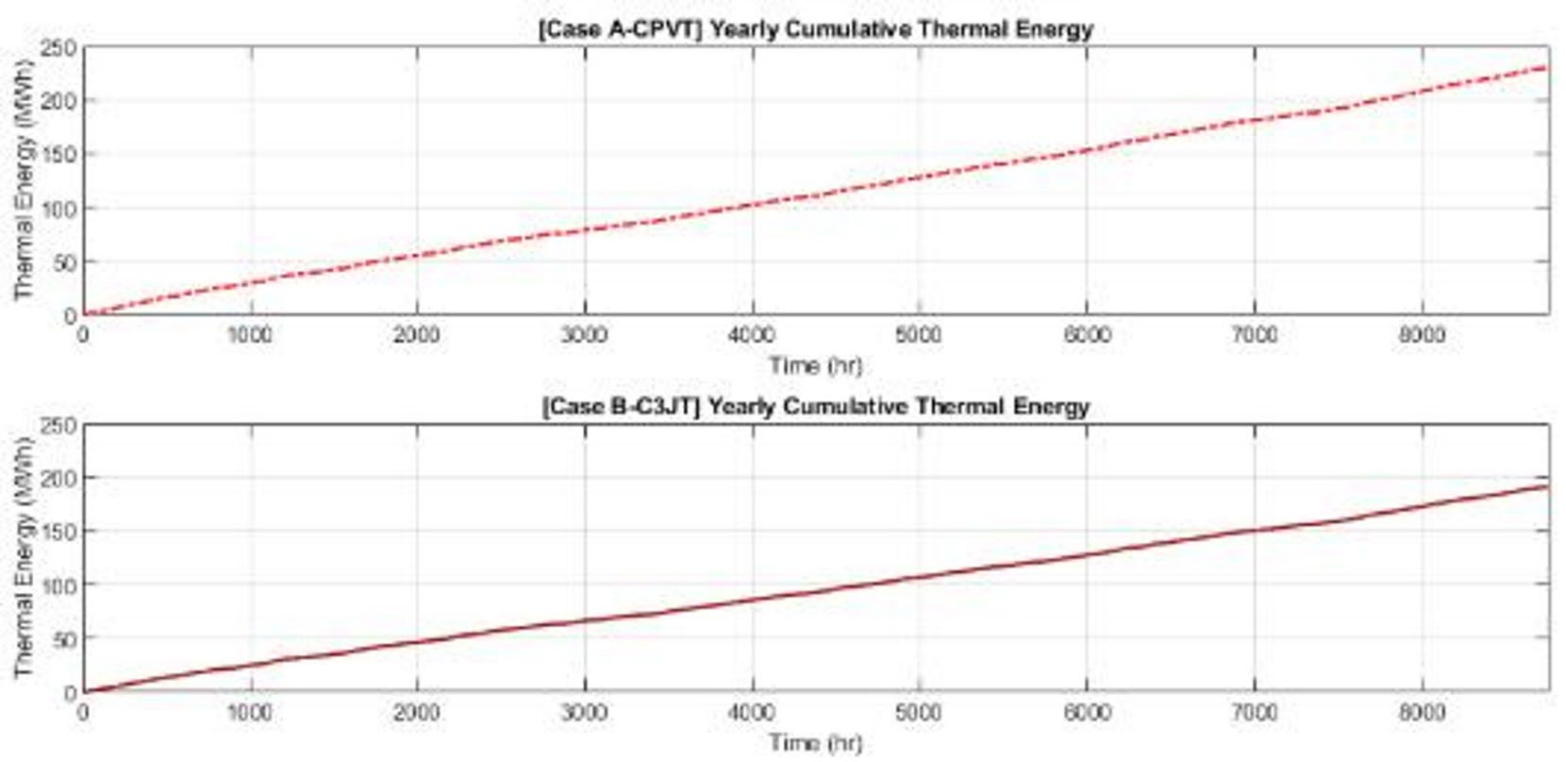
Hourly annual cumulative thermal energy for Cases I-A and I-B at Hurghada.

The total useful thermal energy delivered to the MEE desalination unit is a critical intermediate variable that directly determines freshwater output. From the simulation as shown in Figs. 12 and 13, the useful thermal energy in polygeneration-I-A amounts to 230.0 MWh/yr at Hurghada, 214.5 MWh/yr at Suez, and 219.2 MWh/yr at Port Said. In polygeneration-I-B, these values reduce to 191.3, 177.5, and 180.2 MWh/yr respectively with a consistent reduction of approximately 16.8–17.3% relative to Case A. This reduction is the direct thermodynamic consequence of the C3JT receiver’s higher photoelectric efficiency: since the C3JT converts a larger fraction of incident optical power into electricity with a correspondingly smaller fraction is available as recoverable heat for the MEE unit.

**Fig. 14:**
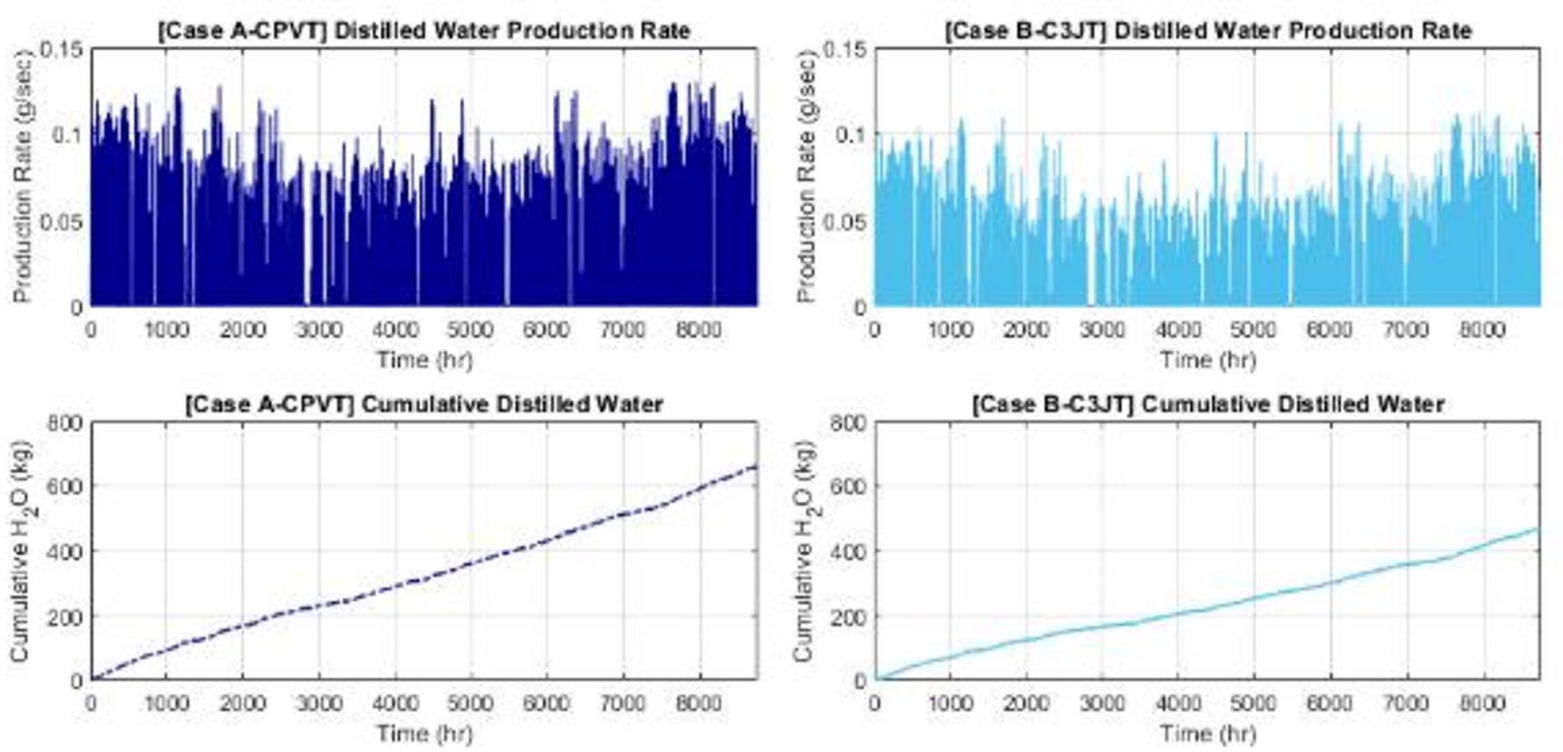
Hourly annual distilled production for Cases I-A and I-B at Hurghada.

The freshwater production results shown in Table 6 reflect this thermal partitioning directly. polygeneration-I-B (C3JT) produces less distilled water than case A (CPVT) with reduction of 29.3%, 31.2%, and 28.9%. These percentage reductions are substantially larger than the thermal energy reduction (16.8–17.3%) because the GOR of the MEE unit is a nonlinear function of the motive steam temperature and flow rate: as the available driving thermal power decreases, the MEE operates at reduced capacity across all six effects, and the combined effect on total distillate is amplified. This nonlinearity is captured within the validated MEE SIMSCAPE block. Within each technology variant, the freshwater production ranking across locations does not follow the DNI ranking. At Hurghada, polygeneration-I-A produces the most freshwater (660.0 t/yr) because it has the highest absolute thermal output. However, Port Said produces more freshwater than Suez in Case A (622.6 vs. 581.1 t/yr) despite having higher DNI than Suez (1,798.8 vs. 1,752.9 kWh m^− 2^ yr^− 1^). This apparent inversion is explained by Port Said’s lower mean ambient temperature (~ 22 °C for Port Said vs. ~ 24 °C for Suez) and thus lower mean seawater inlet temperature, which reduces MEE thermal losses to ambient and improves heat transfer effectiveness in the lower effects (Fig. 14).

#### Hydrogen to solar system efficiency

**Fig. 15:**
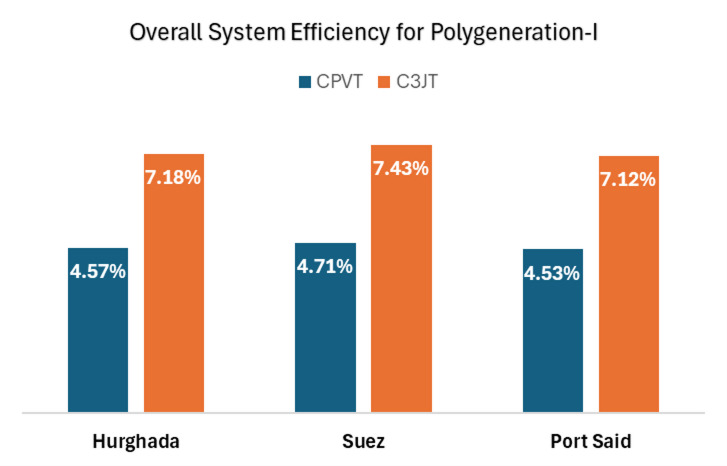
Overall system HHV efficiency for polygeneration-I.

The HHV-based system efficiency values for Polygeneration-I range from 4.53% at Port Said (Case A) to 7.43% at Suez (Case B), as shown in Fig. 15. Across all three locations, the C3JT configurations (Cases I-B) achieve efficiencies approximately 57–58% higher than the corresponding CPVT configurations, driven entirely by the higher photoelectric conversion efficiency of the triple-junction receiver. Within each technology variant, Suez consistently achieves the highest efficiency despite having the lowest DNI among the three locations (1,752.9 kWh m^−2^ yr^−1^). This is explained by Suez having the lowest mean wind speed among the three locations as shown in Table 5, which reduces receiver thermal losses throughout the year, directing a larger fraction of the available solar energy toward useful electrical output and hydrogen production per unit of solar input. Port Said, despite having the lowest mean ambient temperature (21.83 °C) which would otherwise favor efficiency, records a lower efficiency than Suez due to two competing factors: its higher wind speed increases convective receiver losses relative to Suez, and more importantly, it has the fewest annual sunshine hours among the three locations, resulting in fewer system operating hours and less cumulative hydrogen production relative to its total annual solar input.

### Polygeneration-II: hydrogen and electricity

#### Annual hydrogen production and electricity export

The introduction of grid electricity export in polygeneration-II fundamentally restructures the energy flow of the system. As described, the PEM electrolyzer in polygeneration-II is fed exclusively by the MEE distillate stream rather than by external deionized water supply. The electrolyzer therefore only consumes the electrical power required to electrolyze the available distillate flow rate, and all remaining electrical power is exported to the grid. Since electrolysis requires approximately 9 kg of water per kilogram of hydrogen produced, and the MEE distillate flow is thermally limited, the PEM consumes typically less than the total electrical output with the remainder exported to the grid. This explains the order-of-magnitude reduction in annual hydrogen production between Polygeneration-I and Polygeneration-II despite identical solar input and receiver configuration. Table 7 presents the annual results for polygeneration-II.

**Table 7:** Annual hydrogen production and exported electricity for Cases II-A and II-B.

Location	H₂ II-A (kg/yr)	H₂ II-B (kg/yr)	Grid II-A (MWh/yr)	Grid II-B (MWh/yr)
Hurghada	72.6	51.3	18.8	33.2
Suez	63.9	43.9	18.4	32.4
Port Said	68.4	48.6	17.9	31.6

#### Hydrogen to solar system efficiency

**Fig. 16:**
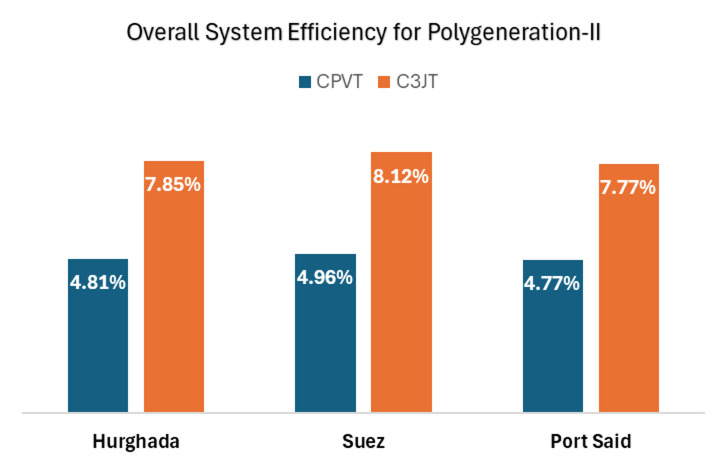
Overall system HHV efficiency for polygeneration-II.

The HHV system efficiency values for polygeneration-II range from 4.77% (Case A, Port Said) to 8.12% (Case B, Suez) as shown in Fig. 16. All cases exceed the corresponding polygeneration-I values at the same location. The efficiency gains from Case I to Case II range from 0.24 to 0.69% points depending on location and technology. This increase is not a thermodynamic improvement in the conversion process itself — the receiver, MEE unit, and PEM electrolyzer operate identically in Cases I and II — but rather a consequence of the HHV efficiency metric of Eq. (25), which credits the numerator with both hydrogen energy content and the electrical energy exported to the grid. By routing the electrical output directly to the grid rather than through the PEM electrolyzer where the system avoids the conversion losses inherent in the electrolysis process and achieves higher net useful energy output per unit of solar input.

### Technoeconomic analysis: levelized cost of hydrogen

#### LCOH results and cross-case comparison

Table 8 presents the LCOH results for all configurations at the three Egyptian locations. The global minimum LCOH of 2.909 USD/kg is achieved by Case II-B at Hurghada, representing a 32.6% reduction relative to the Polygeneration-I CPVT baseline (Case I-A) at the same location.

**Table 8:** LCOH for all system configurations at different Egyptian locations.

Location	LCOH (USD/kg)				Best Architecture	Δ vs. I-A (%)
	I-A	I-B	II-A	II-B		
Hurghada	4.317	3.623	3.441	2.909	II-B	≈ −32.6
Suez	4.525	3.752	3.942	3.441	II-B	≈ −24.0
Port Said	4.523	3.767	4.744	3.686	II-B	≈ −18.5

#### Technology effect: CPVT versus C3JT

Comparing Cases I-A and I-B at fixed architecture (Polygeneration-I), the C3JT receiver delivers LCOH reductions of 16.1% at Hurghada, 17.0% at Suez, and 16.7% at Port Said relative to the CPVT baseline. The same technological effect is observed within Polygeneration-II, where Case II-B consistently outperforms Case II-A at all three locations. The consistency of this reduction across all locations and both architectures confirms that receiver technology selection is the most robust lever for LCOH improvement in this system which is independent of deployment site or polygeneration configuration.

The physical origin of this advantage stems from the spectral, electrical, and thermal mechanisms discussed : the C3JT receiver produces 62–63% more annual electrical output per unit aperture area than the CPVT receiver, driving proportionally higher hydrogen production that spreads the fixed yearly capital cost over a larger hydrogen mass in the LCOH denominator. The MEE freshwater revenue credit and electricity credit remain important, but it is the hydrogen production volume that most strongly determines the LCOH at the scales analyzed here.

#### Topology effect: polygeneration-I versus polygeneration-II

The transition from Polygeneration-I to Polygeneration-II introduces grid electricity export as co-product revenue stream. The effect of this transition on LCOH is strongly location-dependent and is not uniformly beneficial. Grid electricity export only reduces LCOH when the receiver generates sufficient electrical output to overcome the hydrogen production reduction. This economic threshold is met by the C3JT receiver but not by the CPVT receiver confirming that grid export is a viable strategy only when paired with high efficiency triple-junction technology.

At Hurghada, both Cases II-A and II-B improve on their Polygeneration-I equivalent: II-A (3.441 USD/kg) reduces I-A (4.317 USD/kg) by 20.3%, and II-B (2.909 USD/kg) reduces I-B (3.623 USD/kg) by 19.7%. Hurghada’s high DNI (1,872.4 kWh m^−2^ yr^−1^) and long sunshine hours produce the largest absolute electricity export volumes among the three locations, generating grid revenue credits large enough to more than compensate for the dramatic reduction in hydrogen production volume that occurs with the Case II architecture.

At Suez, Case II-B (3.441 USD/kg) improves on Case I-B (3.752 USD/kg) by 8.3%, confirming that the C3JT + electricity export combination is useful. However, Case II-A (3.942 USD/kg) shows a smaller improvement over Case I-A (4.525 USD/kg) of only 12.9%, and the absolute LCOH of Case II-A remains high because the CPVT system exports only 18.4 MWh/yr of electricity — a modest revenue credit that must compensate for the large hydrogen production reduction from 503.0 kg/yr (Case I-A) to 63.9 kg/yr (Case II-A).

**Fig. 17:**
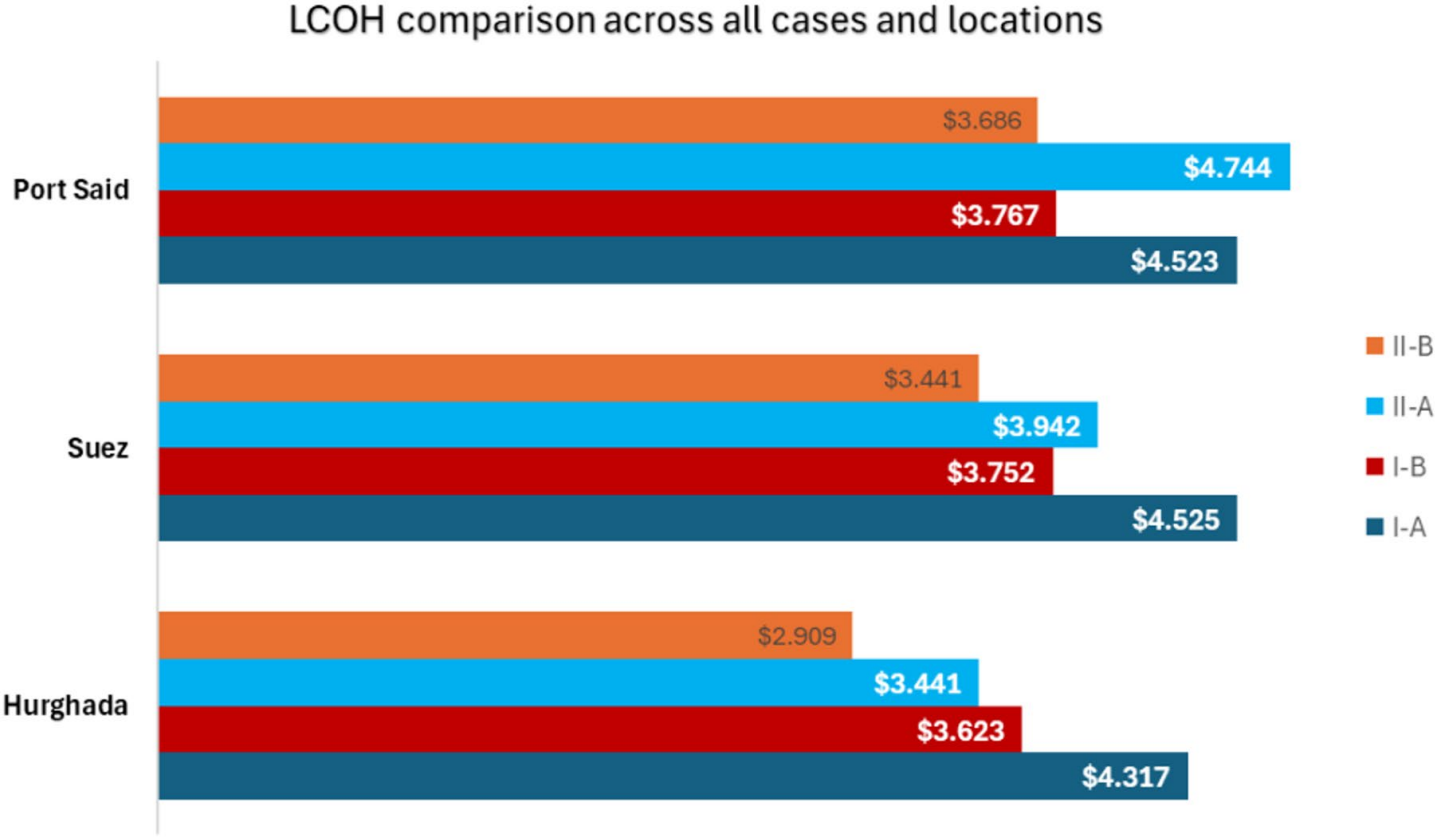
LCOH comparison across all cases and locations.

At Port Said, the most important irregularity in the dataset appears: Case II-A records an LCOH of 4.744 USD/kg, which is higher than its own Case I-A baseline of 4.523 USD/kg as it is the only example in the entire dataset where adding an electricity export pathway increases rather than decreases the LCOH. The explanation lies in the combination of two unfavorable factors at Port Said for the CPVT Case II-A configuration: first, Port Said has the fewest annual sunshine hours among the three locations, producing the smallest grid electricity export volume and therefore the smallest revenue credit; second, the CPVT receiver produces the least electrical power overall, meaning the hydrogen production denominator in the LCOH formula collapses from 496.4 kg/yr in Case I-A to only 68.4 kg/yr in Case II-A. The 17.9 MWh grid electricity revenue is simply insufficient to offset the sensitivity of the LCOH formula to a denominator that has been reduced. This is not an error in the calculation — it is a physically meaningful result that identifies a clear economic boundary condition: grid electricity export is only beneficial for LCOH when the electricity revenue credit is large relative to the capital cost, which requires either high electricity export volume, high electricity tariff, or high receiver electrical output. None of these conditions are met for the CPVT system at Port Said. Case II-B at Port Said (3.686 USD/kg), by contrast, does improve on I-B (3.767 USD/kg) because the C3JT exports 31.6 MWh/yr, generating a revenue credit large enough to overcome the denominator reduction (Fig. 17).

#### Parametric sensitivity analysis

**Fig. 18:**
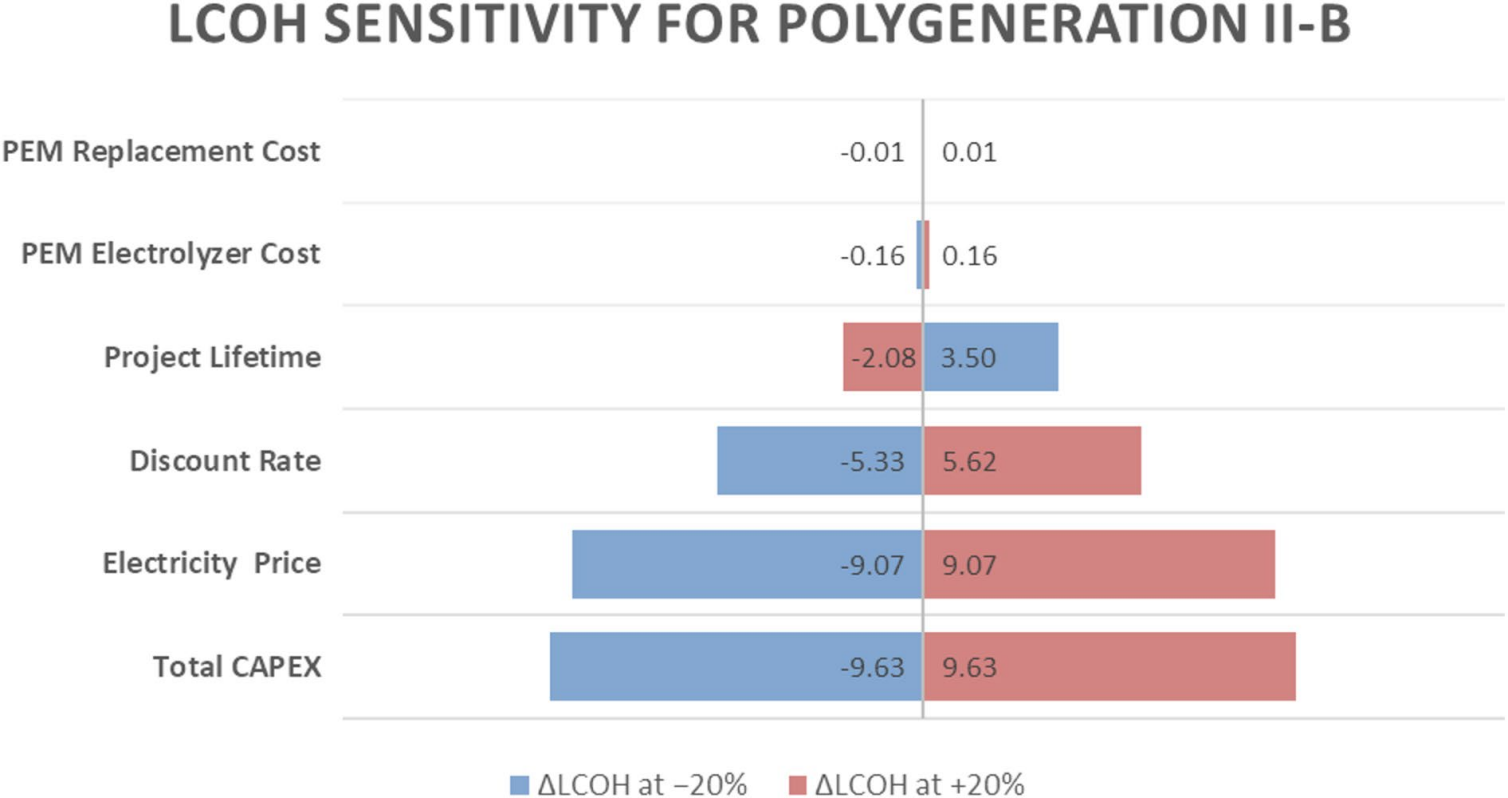
LCOH sensitivity for polygeneration II-B.

A one-way parametric sensitivity analysis was performed for the global minimum LCOH case (Case II-B, Hurghada, base LCOH = 2.909 USD/kg) to evaluate the robustness of the economic conclusions against uncertainties in the key input assumptions. Six parameters were independently varied by ± 20% from their base case values while all other parameters were held constant: (a) total CAPEX, (b) electricity selling price, (c) discount rate, (d) project lifetime, (e) PEM electrolyzer cost, and (f) PEM stack replacement cost. Results are presented in Fig. 18. Total CAPEX and grid electricity selling price are identified as the two dominant parameters, each producing a sensitivity swing across the ± 20% variation range. The discount rate ranks as the third most sensitive parameter reflecting the long project lifetime over which capital costs are recovered. By contrast, PEM electrolyzer capital cost and PEM stack replacement cost have negligible influence on LCOH because the PEM unit represents less than 2% of total system CAPEX at the distributed scale studied.

#### Comparison with published literature

A comparison for LCOH and system efficiency was conducted in Table 9 to position the present research results within the published solar-driven hydrogen and polygeneration studies. The LCOH values achieved in this study (2.909–4.744 USD/kg) are competitive relative to published benchmarks at comparable and larger scales. Hydrogen costs of 39.5–43.1 USD/kg reported in^9^ for off-grid solar-wind hybrid systems in Saudi Arabia without any co-product integration. This order-of-magnitude difference relative to the present results (2.909–4.744 USD/kg) reflects three factors: (i) scale as the systems are small isolated off-grid configurations without economies of scale; (ii) co-product revenue as those systems carry no desalination or electricity export credits, whereas the present LCOH formulation credits all co-product revenues against total project costs; and (iii) solar resource and technology as concentrated triple-junction receivers under Egyptian DNI conditions achieve substantially higher solar-to-hydrogen conversion efficiency than flat-plate PV-wind hybrid systems under Saudi Arabian conditions. The GW-scale studies of Muheiithen et al.^10^ and Al-Ghussain et al.^11^ achieve lower absolute LCOH values of below 1.059 and 1.61 USD/kg respectively but doing so with significant renewable overcapacity and large-scale storage deployment that is fundamentally inapplicable to the distributed scale of the present work. The closest technology analogue is the kilowatt-scale CPV-PEM co-generation pilot of Holmes-Gentle et al.^36^, which reported a system-level solar-to-hydrogen efficiency of 6.6% HHV under real outdoor conditions consistent with the 7.12–7.43% HHV achieved by the C3JT configurations in Polygeneration-I, confirming that the present simulation results are physically realistic. For large-scale renewable hydrogen in Europe, Colangelo et al.^37^ reported an LCOH of approximately 6.37–7.23 USD/kg under European capital cost structures. The global minimum LCOH of 2.909 USD/kg at Hurghada Case II-B represents the only result in this comparison without gigawatt-scale deployment, government subsidy assumptions, or long-range cost projections establishing the economic viability of distributed concentrating solar polygeneration as a practically deployable pathway for green hydrogen production in Egypt.

**Table 9:** Comparison of LCOH and system efficiency against published studies.

Study	Location	Scale	Technology	LCOH (USD/kg)	System Efficiency (% HHV)	Key Distinction
Al-Sharafi et al.^9^	Saudi Arabia (Yanbu)	kW-scale, off-grid	PV + Wind hybrid	39.5–43.1	Not reported	No co-product revenue; HOMER optimization; earliest MENA benchmark
Muhieitheen et al.^10^	NEOM, Saudi Arabia	GW-scale	PV + Wind + Battery	< 1.059	Not reported	Mega-project; 500,000 m³/day desalination; massive grid overcapacity
Al-Ghussain et al.^11^	Jordan	Country-scale GW	PV + Wind + Lithium-ion storage	1.61	Not reported	Country-scale 100% renewable; RO desalination at 0.36 USD/m³
Holmes-Gentle et al.^36^	Laboratory (outdoor)	kW pilot	CPV-PEM (integrated)	Not reported	6.6%	Closest technology analogue; device-level STH 20.3%; no economic analysis
Colangelo et al.^37^	Italy	100–200 MW	PV / Wind	6.37–7.23	Not reported	European capital costs; no co-product revenue; higher LCOH baseline
Present study Poly-I (I-A/I-B)	Egypt: (Hurghada, Suez, Port Said)	Distributed 240 m²	CPVT / C3JT + MEE	3.62–4.52	4.53–7.43%	H₂ + freshwater; year-long hourly dynamic SIMSCAPE simulation
Present study Poly-II (II-A/II-B)	Egypt: (Hurghada, Suez, Port Said)	Distributed 240 m²	CPVT / C3JT + MEE + Grid	2.909–4.744	4.77–8.12%	H₂ + electricity; year-long hourly dynamic SIMSCAPE simulation

## Conclusions

According to the above analysis, the related literature lacks the availability of studies including integration of solar CPVT/C3JT concentrating receivers with PEM electrolysis and MEE thermal desalination on a dynamic basis. This study presented a year-long dynamic simulation comparative analysis of novel solar-driven polygeneration configurations at three Egyptian Red Sea coast locations. Two concentrating solar receiver technologies were evaluated across two polygeneration architectures: Polygeneration-I integrating MEE thermal desalination, and Polygeneration-II additionally introducing electricity export. The following principal conclusions are drawn:

1. The C3JT receiver is the superior technological choice across all configurations and all locations. The GaInP/GaInAs/Ge triple-junction receiver achieves HHV system efficiencies of 7.12–7.43% in Polygeneration-I and 7.77–8.12% in Polygeneration-II, compared to 4.53–4.71% and 4.77–4.96% for the CPVT receiver respectively. This consistent 57–58% efficiency advantage stems directly from the triple-junction cell’s broader spectral absorption and higher photoelectric conversion efficiency, and it translates into a 16–17% LCOH reduction at every location and in every configuration.
2. MEE thermal desalination integration creates real economic value. Diverting receiver thermal output to the MEE unit reduces system-to-hydrogen efficiency as it slightly increases thermal operating losses. However, the freshwater revenue recovers this penalty in all cases, confirming that Polygeneration-I is always economically preferable.
3. Electricity export reduces LCOH, but the benefit is architecture-dependent and location-sensitive. At Hurghada, Polygeneration-II Case II-B achieves the global minimum LCOH of 2.909 USD/kg, a 32.6% reduction relative to the Polygeneration-I CPVT baseline (Case I-A, 4.317 USD/kg). At Suez, Case II-B achieves 3.441 USD/kg (− 24.0%). However, at Port Said, Case II-A records an LCOH of 4.744 USD/kg — higher than its own Polygeneration-I baseline of 4.523 USD/kg — the only example in the dataset where adding grid export increases rather than reduces LCOH. This anomaly is explained by the combination of Port Said’s fewest annual sunshine hours producing the smallest grid export volume and the CPVT receiver’s lowest absolute electrical output, creating a revenue credit too small to compensate for the collapse of the hydrogen production denominator. This result identifies a clear economic boundary condition: grid electricity export only reduces LCOH when the electricity revenue credit is sufficiently large relative to the capital cost structure, a threshold that requires either high electricity export volume, high receiver electrical output, or both.
4. The global minimum LCOH of 2.909 USD/kg is achieved at Hurghada under the Polygeneration-II C3JT configuration (Case II-B). This result is the compound outcome of two contributions of comparable magnitude: Technology effect from upgrading the receiver from CPVT to C3JT, and an architecture effect from adding revenue from electricity export to the MEE polygeneration system.
5. Location selection interacts with architecture choice in a non-trivial way. Hurghada, with the highest DNI and longest sunshine duration, achieves the global minimum LCOH of 2.909 USD/kg and the highest absolute hydrogen production across all configurations. However, its highest mean ambient temperature (25.78 °C) and highest wind speed (5.958 m/s) increase receiver thermal losses relative to the other locations, making it less efficient per unit of solar input than Suez. Suez, despite having the lowest DNI, consistently achieves the highest system efficiency across all cases because its lowest mean wind speed minimizes convective receiver losses throughout the year, directing the largest fraction of solar input toward useful output per unit of resource. Port Said presents the most complex trade-off: its lowest mean ambient temperature would theoretically favor performance, but this advantage is offset by its higher wind speed than Suez and fewest annual sunshine hours, which together reduce cumulative hydrogen production relative to total solar input and make it the least suitable location for grid export architectures when paired with the CPVT receiver. Under the C3JT receiver, however, Port Said remains economically competitive in all configurations.

Future work should investigate the integration of thermal energy storage to enable MEE operation during off-sun hours, thereby increasing annual freshwater production and improving system capacity factor. The sensitivity of Polygeneration-II LCOH to time-of-use electricity tariff structures and renewable energy feed-in tariff policies should also be examined. The inclusion of a comprehensive exergy analysis would provide deeper thermodynamic insight into the irreversibility distribution across the integrated subsystems and is recommended as a valuable extension of the present energetic and economic framework. Construction of an integrated C3JT-PEM-MEE polygeneration prototype at pilot-scale would further consolidate the simulation findings presented in this study.

## Data Availability

All data generated or analyzed during this study are included in this article.
